# Supplemental Lighting Enhances Blueberry Fruit Quality and Flavonoid Accumulation Associated with *VcMYBPA1* Expression in Greenhouses

**DOI:** 10.3390/plants15172671

**Published:** 2026-08-31

**Authors:** Xin Feng, Bingjie Zhou, Jiali Wei, Huiling Wu, Man Cao, Yaqian Zhang, Yaping Wang, Baoshi Guo, Zhixia Hou

**Affiliations:** 1 State Key Laboratory of Efficient Production of Forest Resources, Laboratory for Silviculture and Conservation of the Ministry of Education, Research & Development Center of Blueberry, Beijing Forestry University, Beijing 100083, China; 2 Guangxi Key Laboratory of Forest Ecology and Conservation, Guangxi Colleges and Universities Key Laboratory for Cultivation and Utilization of Subtropical Forest Plantation, College of Forestry, Guangxi University, Nanning 530004, China; 3 Shaanxi Academy of Forestry, Xi’an 710003, China

**Keywords:** *Vaccinium* spp., LED, supplemental lighting, fruit quality, anthocyanin, *VcMYB*

## Abstract

The efficient utilization of light energy is a powerful guarantee to improve the production efficiency of agricultural and forestry facilities. This study investigated the effects of early-morning supplemental lighting on the yield and fruit quality of greenhouse-grown blueberry and examined the physiological and transcriptional responses associated with anthocyanin accumulation. The findings indicate that supplemental lighting significantly promoted fruit development, accelerated maturation, and increased fruit size, weight, sugar, vitamin C content and the antioxidant capacity of the fruits, especially the anthocyanin content. Transcriptomic analysis revealed that, during fruit ripening, the differentially up-regulated genes activated by supplemental lighting primarily involved transmembrane transport functions, membrane components, and redox enzyme activity. Conversely, the down-regulated genes were mainly involved in organic acid metabolism, photosynthesis and photosystem functions. Pathways associated with the up-regulated genes were largely connected to anthocyanin biosynthesis and galactose metabolism. Furthermore, the anthocyanin biosynthesis showed affiliations with several distinct genes (such as *4CL* and *UFGT*) that were differentially up-regulated, along with the *MYB* transcription factor. Notably, in blueberries exposed to supplemental lighting, *VcMYBPA1* showed a strong positive correlation with *UFGT*. Further investigations demonstrated that *VcMYBPA1* was mainly localized in the nucleus, and its overexpression significantly increased the expression levels of *AtDFR*, *AtANS*, and *AtUFGT* in *Arabidopsis*. This study highlights the crucial role of supplemental lighting in enhancing blueberry yield and quality and provides an effective way to further improve the utilization of light-assisted agricultural technologies.

## 1. Introduction

Flavonoids in fruits and vegetables, which are closely linked to light conditions, have garnered significant attention due to their potent antioxidant properties and associated health benefits. Blueberries (*Vaccinium* spp.) are cultivated globally for their nutritional, health, and economic value [1], which is closely associated with their rich flavonoid content, especially anthocyanins, proanthocyanidins (PAs), and flavonols [2].

Light is a key environmental factor influencing plant growth and development, especially in modern facility agriculture [3]. Blueberries are mainly cultivated in open-field production systems, but protected cultivation, including greenhouses and high tunnels, has increasingly been used to reduce climatic risks and extend the production season. Protected cultivation allows partial regulation of temperature, water supply, and light conditions, protects flowers and fruits from frost, rainfall, hail, and strong winds, and can advance flowering and fruit ripening, thereby extending the harvesting and marketing periods. However, the yield advantage of protected cultivation is not universal and depends on cultivar, plant age, pollination, greenhouse structure, and microclimatic management. Although protected environments can promote earlier ripening and reduce weather-related losses, excessive temperatures and reduced incident radiation may adversely affect fruit set, photosynthetic performance, and productivity [4]. Therefore, appropriate regulation of the greenhouse light environment is essential for achieving stable blueberry yield and fruit quality.

In facility cultivation, supplemental lighting strategies involving spectral composition, light intensity, duration, and timing have become important approaches for improving crop productivity and fruit quality. Studies have shown that red light (600–700 nm) and blue light (400–500 nm) not only affect primary metabolism through photosynthesis, but also their spectral ratios and exposure durations synergistically regulate the accumulation of secondary metabolites such as anthocyanins [5]. The response of plants to light spectra varies among species. Red light has been shown to induce anthocyanin synthesis in bilberry (*V. myrtillus* L.) [6] and ‘Red Zaosu’ pears [7], whereas blue light can stimulate anthocyanin accumulation in Chinese bayberry fruit [8] and strawberries [9]. Although both red and blue light can promote plant growth to some extent, numerous studies suggest that monochromatic red or blue light is not the optimal source for maximizing plant growth [10,11,12,13]. For instance, a red–blue light ratio of 1:2 can optimize the growth of pitaya plantlets [10], while a mixed light ratio of 3:7 of blue and red significantly enhances anthocyanin and yield in strawberries [13]. Generally, more light is more conducive to the accumulation of flavonoids such as anthocyanins in plants like Litchi [14] and grapes [15]. In bilberries, compared with a 12 h day length treatment, a 24 h day length results in a higher anthocyanin level in the fruits [16]. In tomatoes, additional LED can reduce the negative impact on yield and quality characteristics caused by double-layer glass and improve fruit quality [17]. Although the effects of light quality on blueberry growth and fruit composition have been investigated, the effects of early-morning supplemental lighting, which simultaneously increases light availability and extends the daily light period, on fruit development, yield, and flavonoid accumulation remain insufficiently understood.

The biosynthesis of anthocyanins and PAs occurs via the flavonoid biosynthetic pathway, regulated by the MBW (MYB-bHLH-WDR) transcription factor complex, which consists of R2R3 myeloblastosis (MYB), basic helix–loop–helix (bHLH), and WD-repeat (WDR) proteins [18,19]. *R2R3 MYB* transcription factors are identified as key regulatory elements in anthocyanin biosynthesis, responding to changes in light-controlled conditions [20]. In grapes, two *R2R3 MYB* transcription factors, *VvMYBA1* and *VvMYBA2*, specifically regulate the expression of the *UFGT* involved in anthocyanin biosynthesis [21]. In grape (*Vitis labruscana*), three MYB-related genes, *VlMYBA1-3*, *VlMYBA1-2*, and *VlMYBA2*, regulate anthocyanin biosynthesis in the berry skin, and their expression patterns are differentially affected by light and temperature conditions [15]. In cultivated strawberry (*Fragaria × ananassa*), light promotes anthocyanin accumulation by inducing *FaMYB10* expression, which subsequently activates anthocyanin biosynthetic genes [22]. Moreover, anthocyanin accumulation was observed in strawberry callus overexpressing *FaMYB10L*, and light treatment enhanced anthocyanin accumulation [23]. These findings indicate that light-responsive MYB transcription factors play important roles in regulating anthocyanin accumulation. However, the transcriptional responses associated with supplemental-light-induced flavonoid accumulation in blueberry fruit remain insufficiently understood.

The efficient use of supplemental light to improve crop productivity and quality remains an important research objective in protected cultivation. Although LED lighting has been widely applied in facility agriculture, previous studies on blueberry have mainly focused on spectral composition or cultivation under fully artificial lighting conditions [24,25]. However, the combined effects of increasing light availability and extending the daily light period through early-morning supplemental LED lighting on blueberry fruit development and flavonoid accumulation under greenhouse conditions remain insufficiently understood. In the present study, scheduled early-morning red–blue LED lighting was used to supplement, rather than replace, natural greenhouse light. Specifically, we aimed to: (1) evaluate its effects on fruit development, estimated yield per plant, ripening, and fruit quality; (2) determine changes in sugar contents, antioxidant-related traits, and flavonoid contents, and identify transcriptomic responses associated with anthocyanin accumulation during fruit ripening; and (3) evaluate the potential role of *VcMYBPA1* in anthocyanin accumulation through expression analysis and heterologous overexpression in *Arabidopsis*. The results provide physiological and molecular evidence for optimizing supplemental light management in greenhouse blueberry production. The results provide phenotypic, biochemical, and transcriptomic evidence for optimizing supplemental light management in greenhouse blueberry production.

## 2. Plant Materials and Methods

### 2.1. Experimental Treatments and Sample Collection

**Figure 1:**
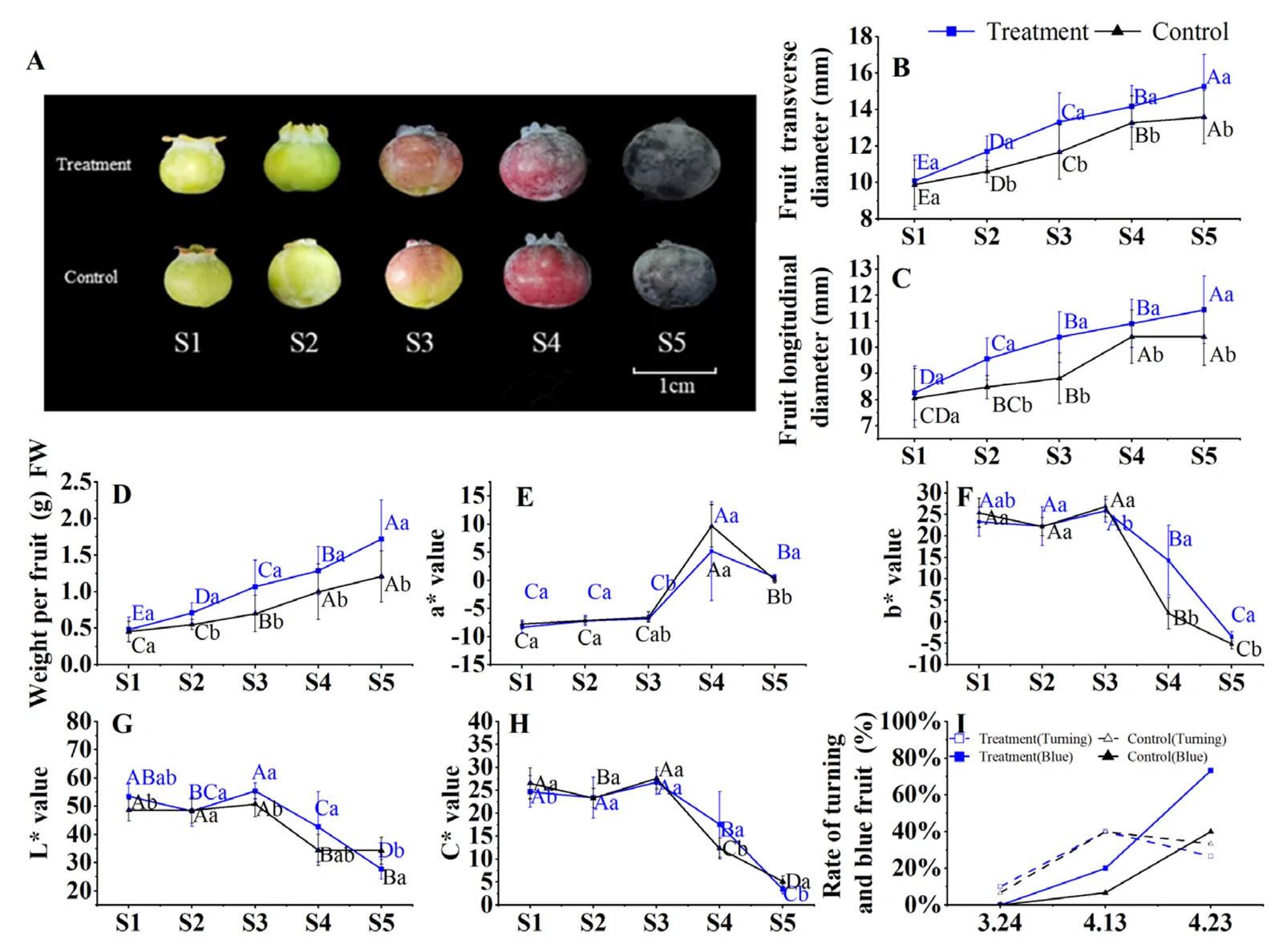
Effects of supplemental lighting treatments on blueberry fruit size, fruit weight, yield and color. (**A**) represents the developmental stages of blueberry (variety ‘M7′) in three stages: green (S1–S2), color transition (S3–S4) and ripening (S5). (**B**–**D**) Transverse diameter, longitudinal diameter, individual fruit weight, respectively, based on measurements of 30 fruits. (**E**,**F**) The a and b values of the fruits, respectively. (**G**,**H**) The L and C values of the fruits, respectively. (**I**) Percentage of fruits at color turning and percentage of fruits at maturity (blue fruit period). The development of 30 fruits was observed at different stages of fruit growth, and the proportion of fruits at maturity and color change stage in each treatment group was calculated among the 30 observed fruits. Upper case letters represent the same treatment and different periods were analyzed by ANOVA (ANOVA, Duncan’s test, *p* ≤ 0.05). Lowercase letters represent the analysis of differences among different treatments within the same period (using *t*-test, *p*-value ≤ 0.05).

The experimental materials were six-year-old northern highbush blueberries (*Vaccinium corymbosum* L.) cultivated in a greenhouse located in the eastern suburbs of Beijing (40°23′ N, 116°47′ E). The blueberry plants were arranged in a north–south orientation with spacing of 1 m × 2 m between plants. To investigate the effect of supplemental lighting on blueberry fruit yield, we conducted a comparative experiment between supplemental light and no supplemental light. Supplemental lighting was applied from 04:00 to 08:00, and the photosynthetic photon flux density of the LED light was 79 μmol m^−2^ s^−1^. Combined with the results of the preliminary experiments and our previous research [26], we selected the light treatment with a higher proportion of blue light (red-to-blue light ratio of 1:5, denoted as 1R5B) as the supplemental light source. This treatment facilitated the growth of blueberry seedlings and significantly increased anthocyanin and soluble sugar contents. To further improve fruit quality, we used the 1R5B ratio as a test group to examine the effects of supplemental lighting in a plant factory on blueberry yield and flavonoid accumulation. Each treatment included three replicates of 10 blueberry plants, totaling 60 plants across both treatments. According to our previous study [27], blueberry fruit development was categorized into five stages: the green fruit stage (S1–S2), the turning stage (S3–S4), and the ripening stage (S5) (Figure 1A). Sampling began at the first fruiting stage on 14 March 2021. Fruits were randomly collected from the upper canopy and periphery of each treatment tree at 7-day intervals. For each sampling, three replicates were taken. The collected fruits were immediately treated with liquid nitrogen and stored at −80 °C for subsequent analysis.

### 2.2. Measurement of Fruit Morphological Characteristics

Three plants were randomly selected from each biological replicate. The number of fruits on each selected plant was recorded, and the estimated yield per plant was calculated by multiplying the mean individual fruit weight by the total number of fruits per plant. The mean value of the three plants was used as one biological replicate. A total of 30 fruits were randomly selected from each treatment and measured. Fruit longitudinal and transverse diameters were measured using vernier calipers, and single fruit weights were measured using a percentage balance with two decimal places. Fruit hardness was determined by a FT-327 fruit hardness tester (Fruit Test™, Rome, Italy), and fruit color was measured using a CS-200 colorimeter (Konica Minolta, Tokyo, Japan) [28]. In color measurements, L* represented black (0) or white (100), a* represented red (+) or green (−), and b* represented yellow (+) or blue (−). Chromaticity values were determined by the formula [(a*)2 + (b*)2]1/2 and represented the overall intensity or saturation of the color. The proportion of fruits in the ripening and turning stages were calculated for each treatment group among the 30 observed fruits, as the rate of turning and blue fruit.

### 2.3. Measurement of Soluble Sugars, Titratable Acids and Chlorogenic Acids

The sugar–acid content of fruit is a critical indicator of fruit ripeness and quality. The soluble sugar content (glucose, fructose and sucrose) of blueberry fruits was determined using an Agilent liquid phase 1260 system (Agilent Technologies, Inc., Santa Clara, CA, USA) equipped with a refractive index detector (RID) and a Zorbax carbohydrate analysis column (250 mm × 4.6 mm, 5 μm, Agilent Technologies, Inc., Santa Clara, CA, USA). The mobile phase consisted of acetonitrile and water in a 9:1 ratio, with isocratic elution at a flow rate of 1 mL min^−1^ and a column temperature of 35 °C [27]. Glucose, fructose, and sucrose were identified by comparing the retention times of sample peaks with those of authentic glucose, fructose, and sucrose standards analyzed under identical chromatographic conditions. Their concentrations were quantified using external standard calibration curves prepared from a series of standard solutions.

The titratable acid (TA) content was determined by sodium hydroxide titration [29]. Specifically, 1 g of each fruit sample was pulverized and extracted using distilled water. Following the addition of two drops of 1% phenolphthalein indicator, the sample underwent titration with a standardized sodium hydroxide solution. The completion of titration was signaled by a persistent pink color in the solution for at least 0.5 min. TA content was computed based on the sample’s acidity level and reported as a percentage (%).

For chlorogenic acid extraction, 0.5 g of frozen fruit powder was mixed with 5 mL of extraction solvent consisting of ultrapure water and 10% (*v*/*v*) acetic acid at a ratio of 85:15 (*v*/*v*). The mixture was sonicated for 30 min in the dark and then centrifuged at 5000× *g* for 10 min [30]. The supernatant is then filtered through a 0.45 μm organic filter membrane for subsequent analysis. An Agilent liquid phase 1260 system (Agilent Technologies, Inc., Santa Clara, CA, USA), equipped with a VWD detector and a Zorbax SB C-18 analytical column (150 mm × 4.6 mm, 5 μm, Agilent Technologies, Inc., Santa Clara, CA, USA), was utilized. The detection wavelength was set at 327 nm, with a sample injection volume of 10 μL. The column temperature was maintained at 25 °C, and a flow rate of 1 ml min^−1^ was employed. The mobile phase consisted of 0.4% phosphoric acid (*v*/*v*) as phase A and methanol as phase B. The elution program was as follows: 0–1 min, 100–90%A; 1–10 min, 90–80%A; 10–15 min, 80–75%A; 15–15.1 min, 75–100%A. Chlorogenic acid was identified by comparing its retention time with that of an authentic chlorogenic acid standard analyzed under the same chromatographic conditions. Its concentration was calculated using an external standard calibration curve.

### 2.4. Extraction and Determination of Flavonoids and Analysis of Antioxidant Activity

Anthocyanin content was determined by the pH difference method [31], approximately 0.5 g of blueberry fruit sample was thoroughly ground in liquid nitrogen and extracted with 1% HCl-CH_3_OH (5 mL). Subsequently, 100 µL of the supernatant was collected after centrifugation and mixed with 4.9 mL of KCl buffer solution at pH = 1 and NaAC buffer solution at pH = 4.5, maintaining stability under 40 °C water bath conditions for 20 min. The reaction mixture was then transferred to a cuvette for spectrophotometric analysis at wavelengths of 520 nm and 700 nm, with distilled water serving as a blank control for calculation purposes. The proanthocyanidin content and flavonol content were determined by the method of Yang [32], with slight modification for the determination of flavonol content. The injection volume was 10 μL, the column temperature was 35 °C, and the flow rate was 1 mL min^−1^. Mobile phase A was 0.2% aqueous acetic acid (*v*/*v*), and mobile phase B was methanol. The elution procedure was as follows: 0–1 min, 24% B; 1–10 min, 28% B; 10–10.1 min, 100% B; and 10.1–14 min, 24% B. Proanthocyanidins and flavonols were identified by comparing the retention times of the sample peaks with those of the corresponding authentic standards analyzed under identical chromatographic conditions. Their contents were quantified using external standard calibration curves.

To determine vitamin C (Vc) content, the 2,6-dichlorophenol-indophenol (2,6-D) assay was employed [29]. This involved the extraction of 10 g of fruit samples using oxalic acid (20 g L^−1^) and subsequent titration with 2,6-D until sustained pink coloration appeared within 15 s. This approach enabled the quantification of ascorbic acid content by evaluating the quantity of 2,6-D utilized during the titration.

The scavenging ability of 2,2-di-(4-tert-octylphenyl)-1-picrylhydrazyl (DPPH), superoxide dismutase (SOD) activity, and malondialdehyde (MDA) content were assessed following the protocol outlined by Yang [32]. For the DPPH assay, 2 g of tissue samples were ground in ethanol (10 mL, 50% *v*/*v*) and then centrifuged at 12,000× *g* for 20 min at 4 °C. For the DPPH radical scavenging activity assay, the extract (0.2 mL) was mixed with DPPH (2.8 mL, 60 mM) and stored in the dark at room temperature for 30 min. The absorbance was recorded at 517 nm. For the SOD activity assay, blueberry tissue (2.0 g) was ground in K phosphate buffer (6 mL, 50 mM, pH 7.3) and then centrifuged at 5000× *g* for 15 min at 4 °C. The supernatant was used to determine SOD activity by assessing its ability to inhibit the reduction of nitroblue tetrazolium (NBT) chloride at 560 nm. The enzyme amount that inhibited 50% of NBT reduction was defined as one unit of SOD (U). In the MDA content determination, 0.5 g of sample was added to 5% TCA solution (5 mL) and ground, then centrifuged to collect the supernatant (2 mL). To the supernatant, 0.67% TBA (2 mL) was added, mixed, and incubated in a 100 °C water bath for 30 min. After cooling to room temperature, the mixture was centrifuged, and the resulting supernatant was used to measure absorbance at wavelengths of 450 nm, 532 nm, and 600 nm. The concentration of MDA was then calculated based on these absorbance values.

### 2.5. Transcriptome Sequencing and Differential Gene Expression (DEGs) Enrichment Analysis

Supplemental lighting significantly influences the development and quality of blueberry fruits. To assess its effects during the ripening phase, we constructed six transcriptome libraries (three biological replicates) from blueberry fruit samples at the ripening stage (S5) under the treatment and control conditions. Libraries were prepared from 1 µg of total RNA utilizing the Illumina^®^ Stranded mRNA Prep, Ligation protocol (San Diego, CA, USA). Sequencing was conducted on the NovaSeq X Plus platform (PE150) with the NovaSeq Reagent Kit (Illumina, Inc., San Diego, CA, USA). The clean reads were aligned to the reference blueberry genome [33] using HISAT2 software.

Gene expression levels were quantified in transcripts per million reads mapped (TPM), and gene abundance was evaluated with RSEM [34]. Differential gene expression analysis (DEGs) was performed using the DESeq2 software package (version 1.24.0), employing the Benjamini/Hochberg method for *p*-value correction. Genes were deemed differentially expressed if |log2(FC)| ≥ 1 and adjusted *p*-value < 0.05. Functional analysis of the differentially expressed genes was conducted using the Majorbio cloud platform, where functional classifications were derived from EggNOG, Gene Ontology (GO), and Kyoto Encyclopedia of Genes and Genomes (KEGG). GO and KEGG pathway enrichment analyses were executed using Goatools software and the Python scipy package (version 1.13.0) [35] (Padjust < 0.05) to further elucidate the biological functions of the DEGs. The heatmap was generated utilizing TBtools (version 2.376) [36].

To explore the potential relationships among calcium signaling, phosphatidylinositol signaling, light signaling, and anthocyanin biosynthesis, candidate genes with higher mean expression in treatment than in control were selected according to GO, KEGG, and gene annotations. Normalized expression values from three B5R and three CK samples were log_2_-transformed, and Pearson correlations were calculated across the six samples. *p* values were adjusted using the Benjamini–Hochberg method, and correlations with |r| ≥ 0.80 and FDR < 0.05 were retained. A target-centered network was constructed around *VcMYBPA1*, *4CL*, and *UFGT*.

### 2.6. Analysis of Relative Expression Levels by qRT-PCR

RNA extraction, reverse transcription to cDNA, and qRT-PCR were conducted as described previously [37]. The gene expression levels were investigated via qRT-PCR using the Prime Script RT-PCR Kit (Vazyme, Beijing, China). The reaction (20 μL) consisted of 10 μL of SYBR, 0.4 μL of forward-specific primer, 0.4 μL of reverse-specific primer, and 1.5 μL of cDNA template. The *GAPDH* and *UBC28* housekeeping genes were used as a reference [38]. The primers were designed using Oligo 7.0 software. The 2^−ΔΔCt^ method was used for relative quantification analysis. All experiments were repeated three times. The specific primers are shown in Supplementary Table S1.

### 2.7. VcMYBPA1 Arabidopsis Transformation and Functional Studies

Based on our analysis, the blueberry gene *VaccDscaff6-snap-gene-261.32* was identified as *VcMYBPA1* (Supplementary Figure S4). The *35S:VcMYBPA1* overexpression vector (vector 3302Y) was constructed using homologous recombination with the ClonExpress Ultra One-Step Cloning Kit (Vazyme, Nanjing, China). The primers used for this construction are listed in Supplementary Table S1. The *VcMYBPA1* gene was introduced into *Arabidopsis thaliana* via the floral dip method [39], and transgenic lines overexpressing *VcMYBPA1* were screened using herbicides. Transgenic lines were further confirmed by PCR amplification using gene-specific primers. The phenotypes of the stabilized T3 generation of transgenic *Arabidopsis* were compared with those of wild-type Columbia *Arabidopsis*. Branch length was measured at 30 days post-germination when the branching rate exceeded 50% [40]. Additionally, the expression levels of structural genes associated with anthocyanin and proanthocyanidin biosynthesis (*AtDFR*, *AtANS*, *AtUFGT*, *AtANR*) were quantified at fruit ripening (60 days) using qRT-PCR. The primers used for qRT-PCR are listed in Supplementary Table S1.

Anthocyanin content in *Arabidopsis* was determined by weighing 0.1 g of fresh plant material from uniformly grown plants, grinding the tissue in liquid nitrogen, and extracting anthocyanins in 0.6 mL of methanol containing 1% concentrated hydrochloric acid. The extract was incubated overnight at 4 °C, followed by the addition of 400 µL of chloroform and 400 µL of distilled water. After mixing and standing for 10 min, the supernatant was centrifuged, and absorbance values at 530 nm and 657 nm were recorded. Anthocyanin content was calculated using the formula (A530 − 0.25 × A657)/fresh weight (g), with three biological replicates per experiment [41]. Proanthocyanidin content was determined using a commercial kit (Stock No. A144-1-1; sourced from Nanjing, China). This kit is based on the principle that under acidic conditions, resorcinol on the A-ring of plant proanthocyanidins undergoes a condensation reaction with vanillin to produce colored compounds with a characteristic absorption peak at 500 nm. Absorbance values at 500 nm were measured, and proanthocyanidin content was calculated accordingly.

For subcellular localization analysis, the *VcMYBPA1–GFP* fusion construct and the empty vector expressing free GFP were transiently expressed in leaves of transgenic tobacco plants stably expressing a red fluorescent NLS nuclear marker via *Agrobacterium*-mediated infiltration [42]. GFP and NLS fluorescence signals were detected using a TCS SP8 confocal laser-scanning microscope (Leica, Wetzlar, Germany).

### 2.8. Statistical Analyses

The study employed a completely randomized design with three replicates for both measurement and analysis. Statistical evaluation was performed using SPSS 26.0 (SPSS Inc., USA), and the *t*-test and Duncan’s test were used to assess significant differences (*p* ≤ 0.05). Correlation analysis was conducted using the Pearson correlation coefficient. The figures presented in this study were generated using Origin and Excel (version 2019).

## 3. Results

### 3.1. Supplemental Lighting Significantly Increases Fruit Yield and Promotes Early Ripening

In the experiments, the longitudinal and transverse diameters of blueberry fruits gradually increased over time (Figure 1A). Notably, the transverse and longitudinal diameters of fruits in the treatment group were significantly larger than those in the control group, especially after the S2 stage (Figure 1B,C). Additionally, the single fruit weight in the treatment group was significantly greater than that in the control group (Figure 1D). Supplemental lighting significantly increased the estimated yield per plant from 2.33 ± 0.37 kg plant^−1^ in the control group to 4.59 ± 0.31 kg plant^−1^ in the treatment group, corresponding to an increase of approximately 97.0%. Thus, the estimated yield under supplemental lighting was approximately 1.97-fold that of the control (Supplementary Figure S1). Regarding fruit coloration (Figure 1E–H), both brightness (L-value) and saturation (C-value) gradually decreased as the fruits matured. At the ripening stage (S5), the fruits primarily displayed blue-purple and red-purple colors, with color values ranging from 25 < L < 35, 0 < a < 1, −6 < b < −3, and 3 < C < 6. From 24 March–23 April, a higher percentage of blue fruit was found in the treatment group (Figure 1I), suggesting that supplemental lighting accelerated blueberry fruit development. Moreover, fruits receiving supplemental lighting reached the coloring and ripening stages approximately 5 days earlier than those in the control group (Figure 1I). Collectively, these findings suggest that supplemental lighting treatment had a significant effect on blueberry fruit enlargement and yield increase.

### 3.2. Supplemental Lighting Increases Blueberry Sugar Content, Changes Fruit Acidity and Improves Antioxidant Capacity

**Figure 2:**
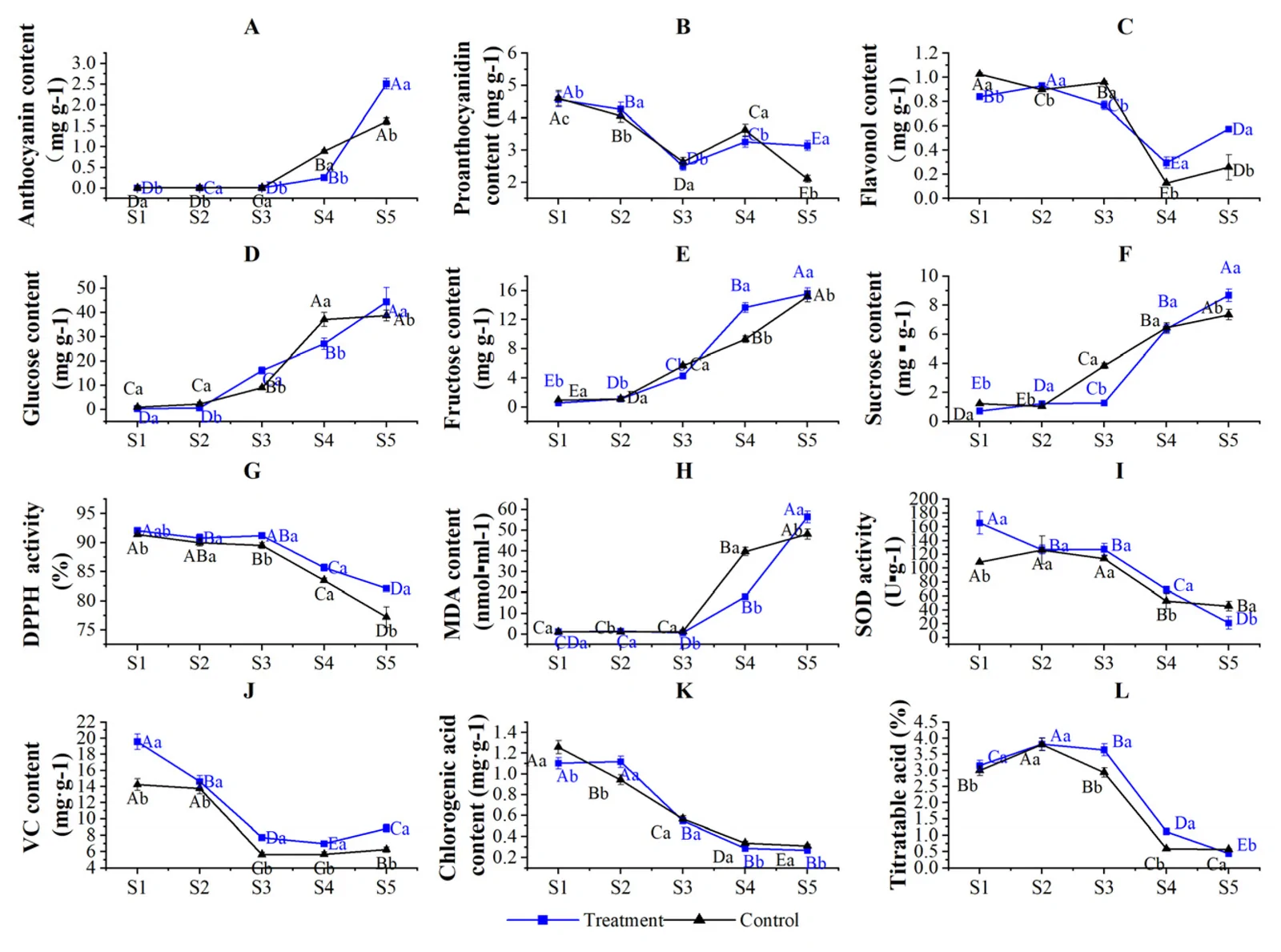
Effects of supplemental lighting treatments on flavonoid content, sugar content and antioxidant capacity in blueberries. (**A**) Anthocyanin content. (**B**) Proanthocyanidin content. (**C**) Flavonol content. (**D**) Glucose content. (**E**) Fructose content. (**F**) Sucrose content. (**G**–**L**) DPPH scavenging capacity, MDA content, SOD activity, Vc content, chlorogenic acid content and titratable acid content, respectively. Lowercase letters indicate analysis of differences between treatments in the same period (*p* ≤ 0.05 using *t*-test). Uppercase letters indicate analysis of variance between different periods of the same treatment (using ANOVA, Duncan’s test, *p* ≤ 0.05). Data means ± SE (*n* = 3). Error bars in the graphs indicate the standard error of three replicates.

During blueberry fruit development, anthocyanin and soluble sugar contents generally increased, whereas proanthocyanidin, flavonol, chlorogenic acid, vitamin C, DPPH radical scavenging activity, and SOD activity showed overall decreasing trends (Figure 2). Anthocyanin content remained low from S1 to S3, began to increase at S4, and accumulated rapidly at S5. At the ripening stage, anthocyanin content was significantly higher in the supplemental light treatment than in the control (Figure 2A).

Proanthocyanidin and flavonol contents generally decreased as the berries matured. However, supplemental lighting maintained higher levels of these compounds during the late developmental stages, particularly at S4 and S5 (Figure 2B,C). Glucose, fructose, and sucrose contents increased progressively during fruit development, although their responses to supplemental lighting varied among developmental stages. At S5, the supplemental light treatment showed higher glucose and sucrose contents than the control, whereas fructose contents were similar between the two treatments (Figure 2D–F). Naturally, during fruit ripening, DPPH radical scavenging activity, SOD activity, Vc, chlorogenic acid, and titratable acid contents decreased, while MDA content increased, indicating a decline in antioxidant capacity with fruit development (Figure 2G–L). However, throughout fruit development (S1–S5), the treatment group showed significantly higher DPPH and Vc contents compared to the control group (Figure 2G,J). From the turning stage to ripening (S3–S5), MDA content was significantly higher in the treatment group (Figure 2H), particularly at the ripening stage (S5). In contrast, SOD content decreased significantly in the treatment group during S3–S5 and was significantly lower than the control at the ripening stage (S5) (Figure 2I). Chlorogenic acid content progressively decreased from S1 to S5, and the two treatments exhibited similar developmental trends, with only limited differences between them (Figure 2K). Titratable acidity increased during the early developmental stages, reached a relatively high level at S2, and subsequently declined sharply. Supplemental lighting maintained higher titratable acidity than the control during the later stages of fruit development (Figure 2L). Overall, supplemental lighting promoted anthocyanin and sugar accumulation and altered several antioxidant-related and organic acid traits during blueberry fruit ripening.

### 3.3. Transcriptome Analysis

**Figure 3:**
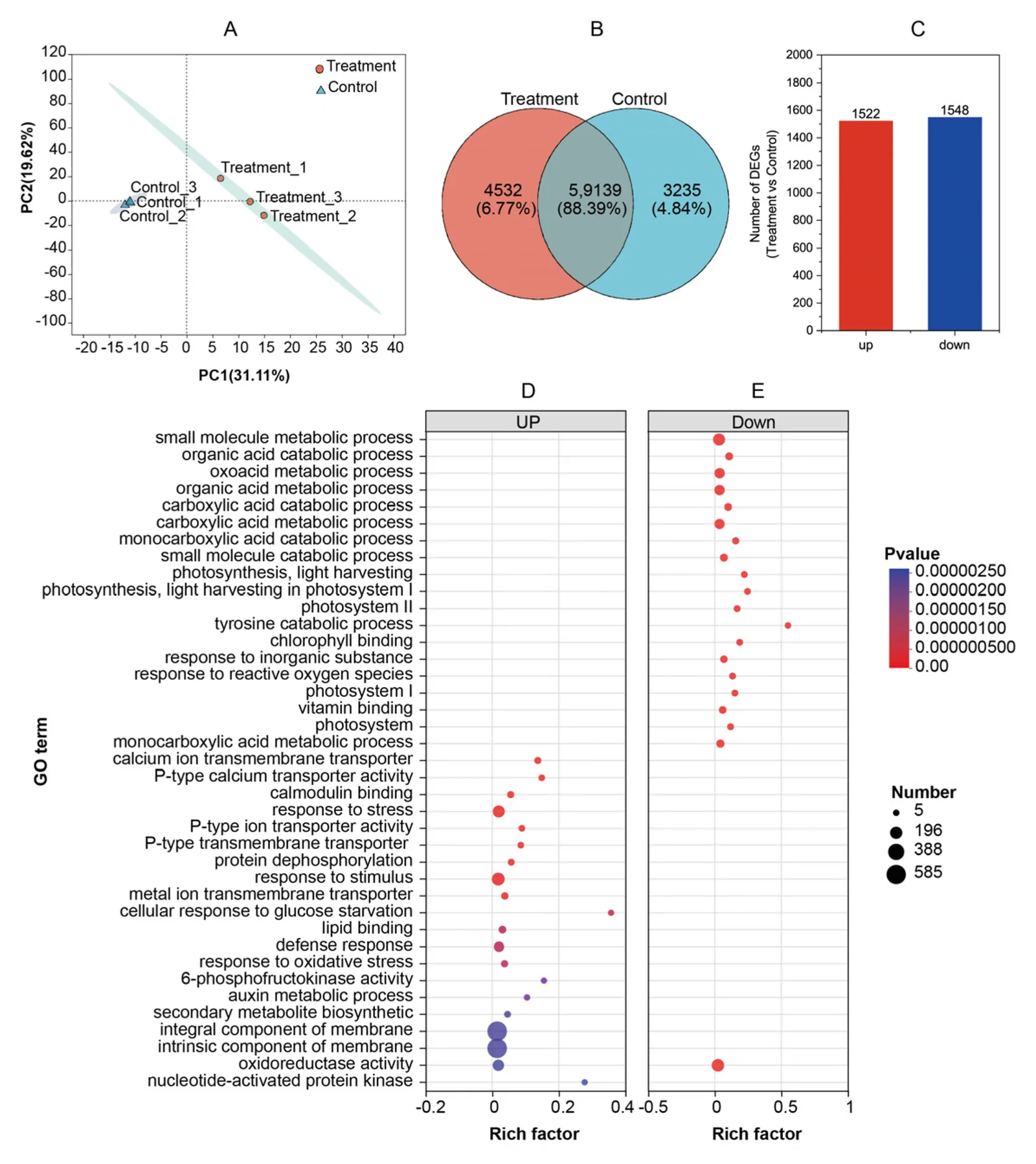
Transcriptome analysis and GO enrichment analysis. (**A**) represents PCA analysis of the transcriptome; (**B**) represents Venn analysis of co-expressed and specifically expressed genes between treatment and control groups; (**C**) represents differential gene analysis of treatment vs. control group; (**D**,**E**) represent the results of GO enrichment analysis of differentially up-regulated and down-regulated genes, respectively.

To further elucidate the effects of supplemental lighting on blueberry fruit ripening, we conducted transcriptome sequencing analysis on blueberry fruits from the treatment and control groups at the S5 stage. The transcriptome sequencing of six samples was completed, with Clean Data exceeding 6.57 Gb for each sample, and the percentage of Q30 bases above 95.65% (Supplementary Table S2). The Clean Reads of all samples were aligned to the reference genome, with an alignment rate ranging from 92.96% to 93.11% (Supplementary Table S3). Correlation analysis (Supplementary Figure S2) and principal component analysis (Figure 3A) indicated good biological reproducibility (three replicates). A total of 63,671 genes were identified in the treatment group and 62,374 genes in the control group, with 59,139 genes commonly identified (Figure 3B). Differential gene analysis revealed a total of 3070 differentially expressed genes (DEGs), including 1522 up-regulated and 1548 down-regulated genes (Figure 3C).

### 3.4. Analysis of Differential Gene Enrichment

GO enrichment analysis of differentially up-regulated and down-regulated genes was performed separately (Figure 3D,E). Up-regulated DEGs were primarily enriched in biological processes such as calcium ion transmembrane transporter activity, P-type calcium transporter activity, calmodulin binding, P-type ion transporter activity, metal ion transmembrane transporter activity, lipid binding, 6-phosphorothioate lipid binding, 6-phosphofructokinase activity, and oxidoreductase activity. These functions involve multiple transmembrane transporters and oxidoreductase activities. In terms of cellular components, up-regulated DEGs were mainly associated with integral and intrinsic membrane components. For molecular functions, up-regulated DEGs were involved in responses to stress, protein dephosphorylation, response to stimuli, and auxin metabolic processes. Down-regulated DEGs were primarily enriched in biological processes related to small-molecule metabolism, organic acid catabolism, oxoacid metabolism, carboxylic acid catabolism, and photosynthesis. Cellular components were mainly related to photosystem function, while molecular functions were primarily chlorophyll binding, oxidoreductase activity, and vitamin binding.

**Figure 4:**
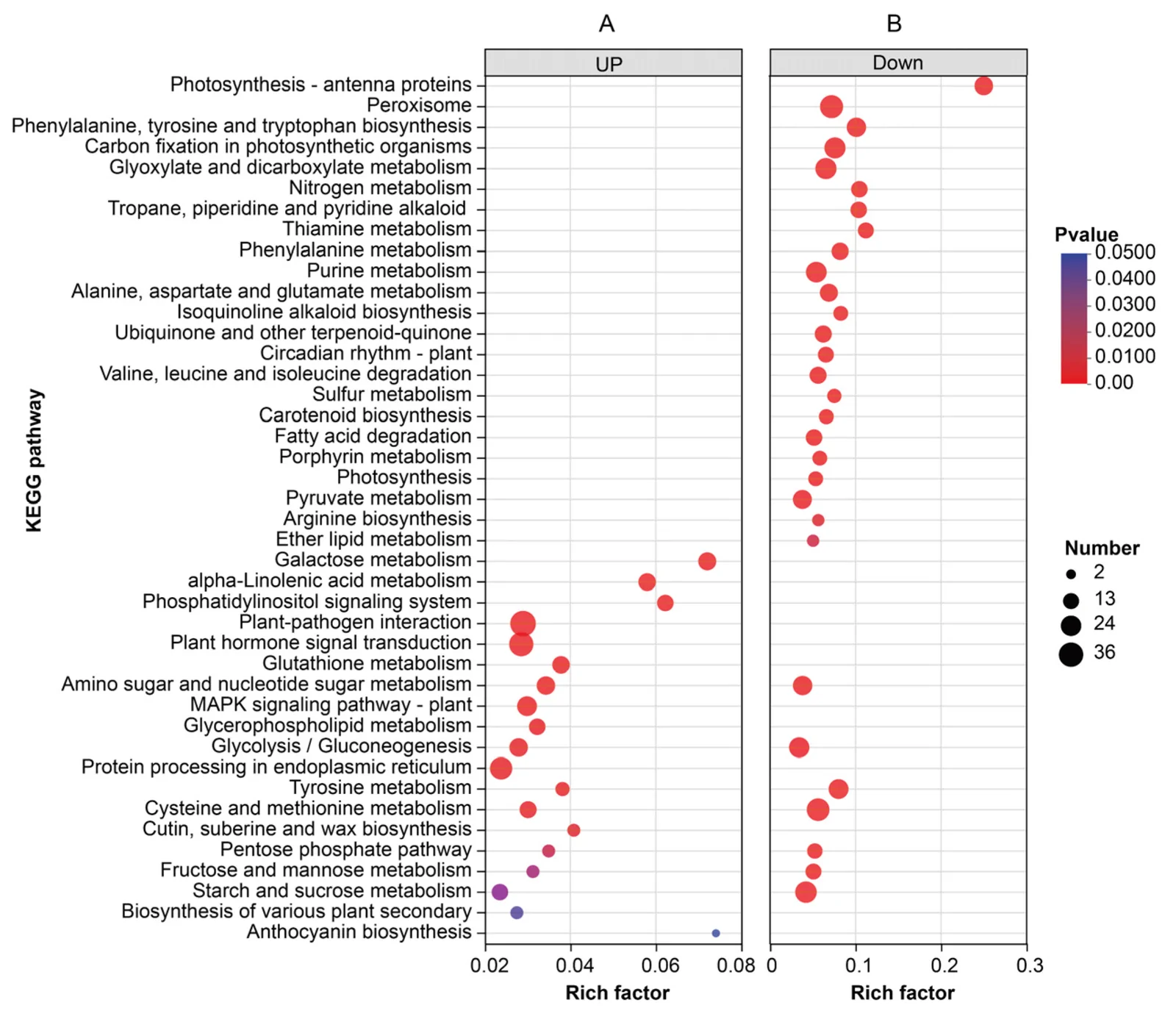
Differential gene KEGG enrichment analysis. (**A**) represents the results of KEGG enrichment analysis of differentially up-regulated genes in the treatment vs. control groups. (**B**) shows the results of KEGG enrichment analysis of differentially down-regulated genes in the treatment vs. control groups.

KEGG enrichment analysis showed that up-regulated DEGs were enriched in pathways such as galactose metabolism, alpha-linolenic acid metabolism, phosphatidylinositol signaling system, plant hormone signal transduction, starch and sucrose metabolism, anthocyanin biosynthesis (Figure 4A). Down-regulated DEGs were enriched in pathways involving phenylalanine, tyrosine, and tryptophan biosynthesis, tyrosine metabolism, and phenylalanine metabolism (Figure 4B). These findings suggest that supplemental lighting treatments significantly up-regulated phytohormone-related synthesis and signaling genes, as well as anthocyanin biosynthesis genes, thereby affecting blueberry fruit quality.

Up-regulated genes were significantly enriched in calcium transport-related processes and the phosphatidylinositol signaling pathway. To further explore the potential links of these pathways with light signaling and anthocyanin biosynthesis, we constructed a candidate pathway–gene–correlation network using normalized expression values from three treatment and three control samples. Candidate genes were assigned to four functional modules—calcium-related processes, phosphatidylinositol signaling, light signaling, and anthocyanin biosynthesis and transcriptional regulation—according to their GO, KEGG, and gene annotations. A total of 94 candidate genes with higher mean expression in treatment than in control were identified, including 61 calcium transport/calmodulin-related genes, 16 phosphatidylinositol-signaling genes, six light-signaling genes, and 14 anthocyanin structural or candidate regulatory genes (Supplementary Figure S3 and Supplementary Table S4).

The target-centered network constructed around *VcMYBPA1*, two *4CL* genes, and two *UFGT*-related genes revealed multiple strong expression associations with calcium transport/calmodulin-related, phosphatidylinositol-signaling, light-signaling, and other *MYB* candidate genes that remained significant after Benjamini–Hochberg correction (Supplementary Figure S3 and Supplementary Table S4). These results indicate coordinated expression among signaling-related and anthocyanin-associated genes under supplemental lighting and provide exploratory evidence for their potential functional convergence.

### 3.5. Identification of Candidate Genes Associated with Anthocyanin Accumulation

**Figure 5:**
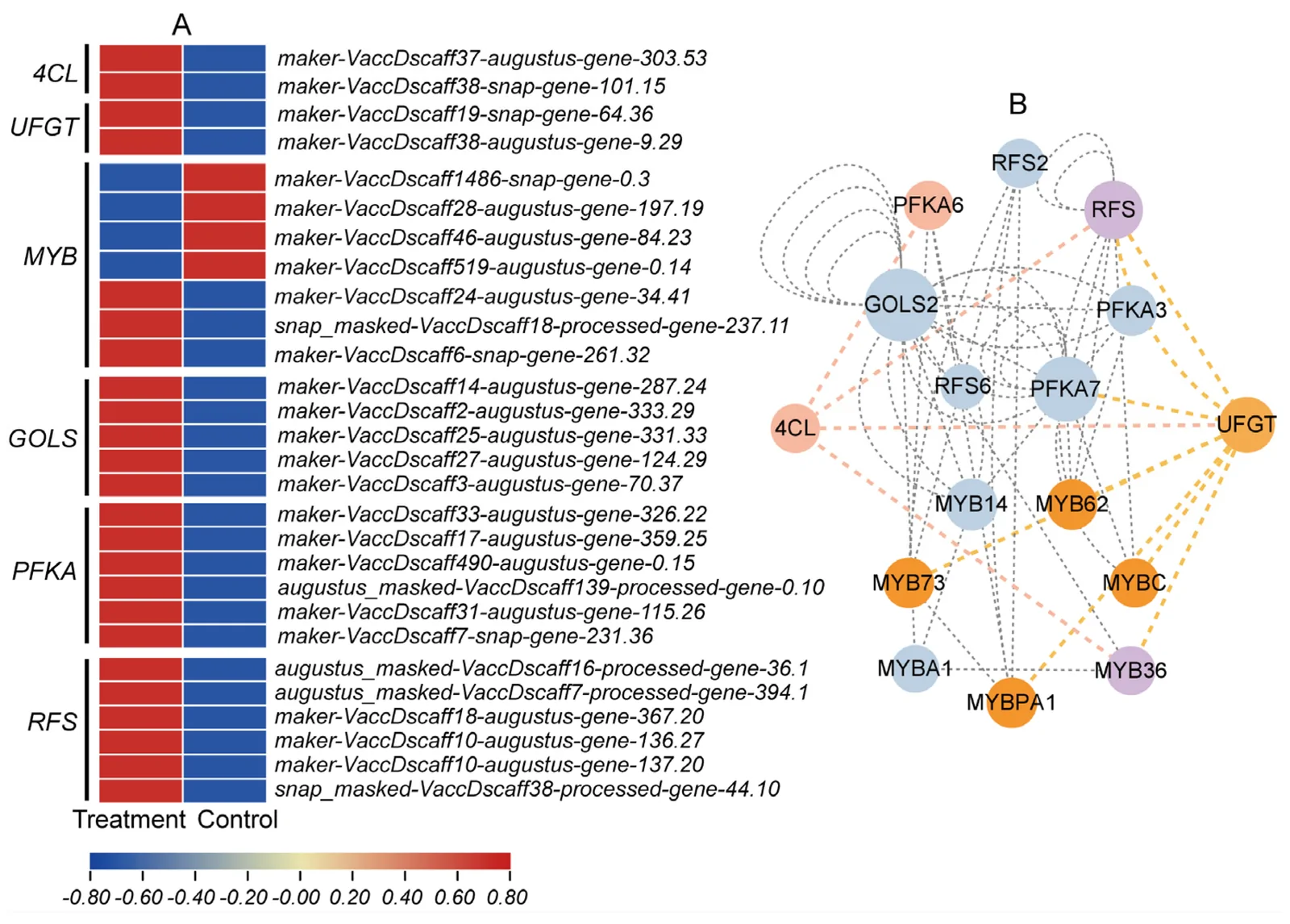
Anthocyanin biosynthesis-associated DEGs. (**A**) Heatmap showing the expressions of differentially expressed genes. (**B**) Expression correlation analysis, where orange represents expression correlation with *UFGT*, pink represents expression correlation with *4CL*, and purple represents expression correlation with both *UFGT* and *4CL*.

During blueberry fruit ripening, the treatment group exhibited a significant increase in anthocyanin and proanthocyanidin content. KEGG enrichment analysis revealed that differentially expressed genes in the galactose metabolism and anthocyanin biosynthesis pathways were significantly enriched. Further transcriptome analysis identified several differentially expressed genes related to anthocyanin synthesis, including *4CL* and *UFGT* genes (Figure 5A). Since the sugar moiety of some anthocyanidins is galactose, their formation depends on UDP-galactosyltransferase. We analyzed the galactose metabolism pathway and identified 17 differentially up-regulated genes, including five *galactoside synthase (GOLS)* genes, six galactoside-sucrose galactosyltransferase (*RFS)* genes, and six ATP-dependent 6-phosphofructokinase (*PFKA*) genes (Figure 5A). Additionally, R2R3 MYB transcription factors have been implicated in anthocyanin synthesis in blueberries [43,44]. In this study, we identified four differentially down-regulated MYB genes and three differentially up-regulated MYB genes (Figure 5A). Through Swiss-Prot annotation and NCBI homology search methods, these seven genes were precisely identified, and finally, the *MYBPA1*, *MYBA1*, *MYBC2.1*, *MYB36*, *MYB62*, *MYB14*, and *MYB73* genes were identified (Supplementary Figure S4). Considering that MYBA-type R2R3 transcription factors have always been regarded as key regulatory elements in anthocyanin biosynthesis [45], the identification of *MYBPA1* and *MYBA1* as members of this MYBA-type transcription factor category provides key clues for further study of the molecular regulatory mechanisms underlying the impact of supplemental lighting on anthocyanin synthesis in blueberries.

To further analyze the relationship among galactose, anthocyanin and *MYB*, expression correlation analysis was conducted on the above-mentioned differentially expressed genes (Figure 5B). The results show that there was an expression correlation between the *4CL* and *RFS*, *PFKA6*, and *MYB36* genes, while *UFGT* was correlated with the *MYBPA1*, *MYB73*, *MYB62*, *MYBC*, *MYB36* and *RFS* genes. There was also an expression correlation between *4CL* and *UFGT*.

Given that anthocyanin biosynthesis is commonly regulated by the MYB–bHLH–WDR complex [44], we further screened the transcriptomic dataset for candidate bHLH and WDR family members. Annotation-based screening of the transcriptomic dataset identified 15 bHLH and 32 WDR family candidates. Four bHLH and 12 WDR candidates showed higher mean expression in treatment than in control. One *BHLH42* candidate (*maker-VaccDscaff19-augustus-gene-381.30*) contained bHLH-MYC_N and HLH domains, and its GO annotation included “positive regulation of anthocyanin biosynthetic process.” Because *TT8* belongs to the bHLH42 group, we cautiously classified this gene as a *BHLH42/TT8-like* candidate. However, its mean expression was slightly lower in the treatment group than in control (log_2_FC = −0.175). Based on the available annotations, no candidate could be confidently identified as a *GL3-*, *EGL3-*, or *TTG1*-like ortholog (Supplementary Table S5).

**Figure 6:**
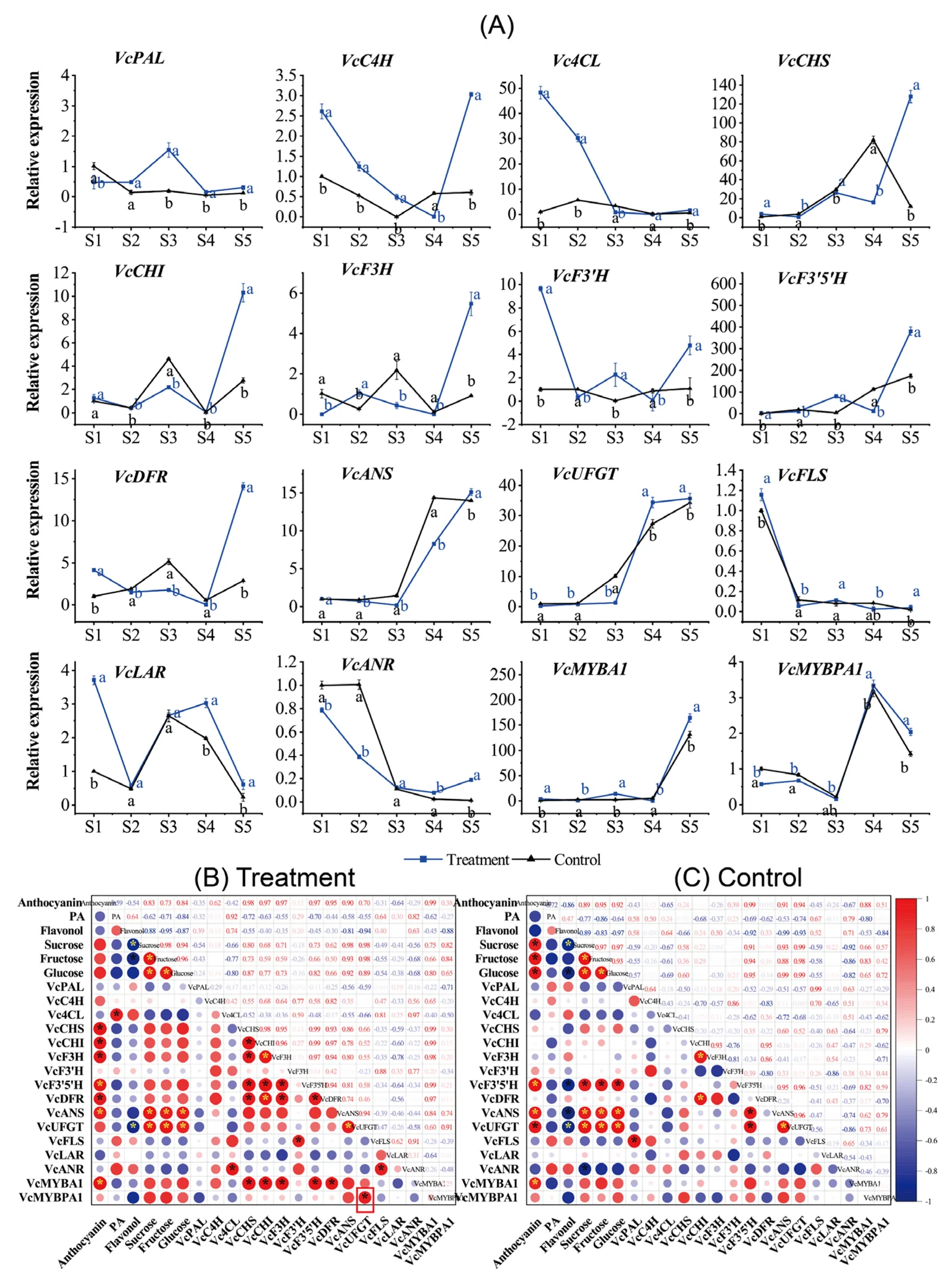
Differential expression analysis of genes involved in flavonoid metabolic pathways during blueberry development in treatment and control groups. (**A**) represents the qRT-PCR results of genes related to the flavonoid metabolism pathway and the MYB gene in blueberry development under different treatments. *VcPAL*, phenylalanine ammonia-lyase; *VcC4H*, cinnamate-4-hydroxylase; *Vc4CL,* 4-coumarate: CoA ligase; *VcCHS*, chalcone synthase; *VcCHI*, chalcone isomerase; *VcF3H*, flavanone 3-hydroxylase; *VcDFR*, dihydroflavonol reductase; *VcF3′H*, flavonoid 3′-hydroxylase; *VcLAR*, leucoanthocyanidin reductase; *VcANR*, anthocyanidin reductase; *VcANS*, anthocyanidin synthase; *VcUFGT*, flavonoid 3-O-glucosyl transferases; *VcF3′5′H*, flavonoid 3′,5′-hydroxylase; *VcFLS*, flavonol synthase. Lowercase letters indicate significant differences in the mean values of different treatments in the same period (*t*-test was used, *p* ≤ 0.05). Error bars in the figure indicate the standard error of three replicates. (**B**,**C**) represent the correlation analysis of the treatment and control groups, respectively, using Pearson for correlation analysis, bilateral test, * represents *p* ≤ 0.05.

To further investigate the effect of supplemental lighting on the synthesis of anthocyanins in blueberries, we conducted qRT-PCR analysis on the key synthase genes and *MYBPA-type* genes in the flavonoid pathway (Figure 6A). Genes encoding key enzymes in the phenylpropanoid pathway and flavonoid pathway, including *VcPAL*, *VcC4H*, *Vc4CL*, *VcCHS*, and *VcCHI*, showed higher expression levels in the S1-S3 stages of the treatment group, while *VcPAL*, *VcC4H*, *Vc4CL*, *VcCHS*, and *VcCHI* showed increased expression levels in the S5 stage. With the ripening of the fruit, the expression levels of *VcF3′5′H*, *VcANS*, *VcUFGT*, and *VcMYBA1* gradually increased, and in both the treatment group and the control group, *VcF3′5′H*, *VcANS*, *VcUFGT*, and *VcMYBA1* were significantly positively correlated with anthocyanin content (Figure 6B,C). In the treatment group, the expression level of *VcMYBA1* was higher than that in the control group, especially in the S3 and S5 stages, and in both the treatment group and the control group, *VcMYBA1* was significantly positively correlated with anthocyanin content. Currently, there is evidence that stable overexpression of *VcMYBA1* in blueberries is sufficient to increase the content of anthocyanins [46], indicating that *VcMYBA1* can increase anthocyanin content. In contrast, genes related to the flavonol pathway (*VcFLS*) and proanthocyanidin pathway (*VcANR*) showed a decreasing trend from the green fruit stage to the mature stage (S1–S5). In the correlation, *VcFLS* and *VcANR* were positively correlated with flavonol and proanthocyanidin content, respectively, in both the treatment group and the control group. Importantly, correlation analysis of qRT-PCR data throughout blueberry development indicated that *VcUFGT* expression was correlated with *VcMYBPA1* expression in both the treatment and control groups. These results suggest that *VcMYBPA1* is a candidate MYB transcription factor associated with *VcUFGT* expression and anthocyanin accumulation under supplemental lighting. Therefore, VcMYBPA1 was selected for further functional characterization in a heterologous system.

### 3.6. Heterologous Overexpression of VcMYBPA1 Enhances Anthocyanin Accumulation in Arabidopsis

**Figure 7:**
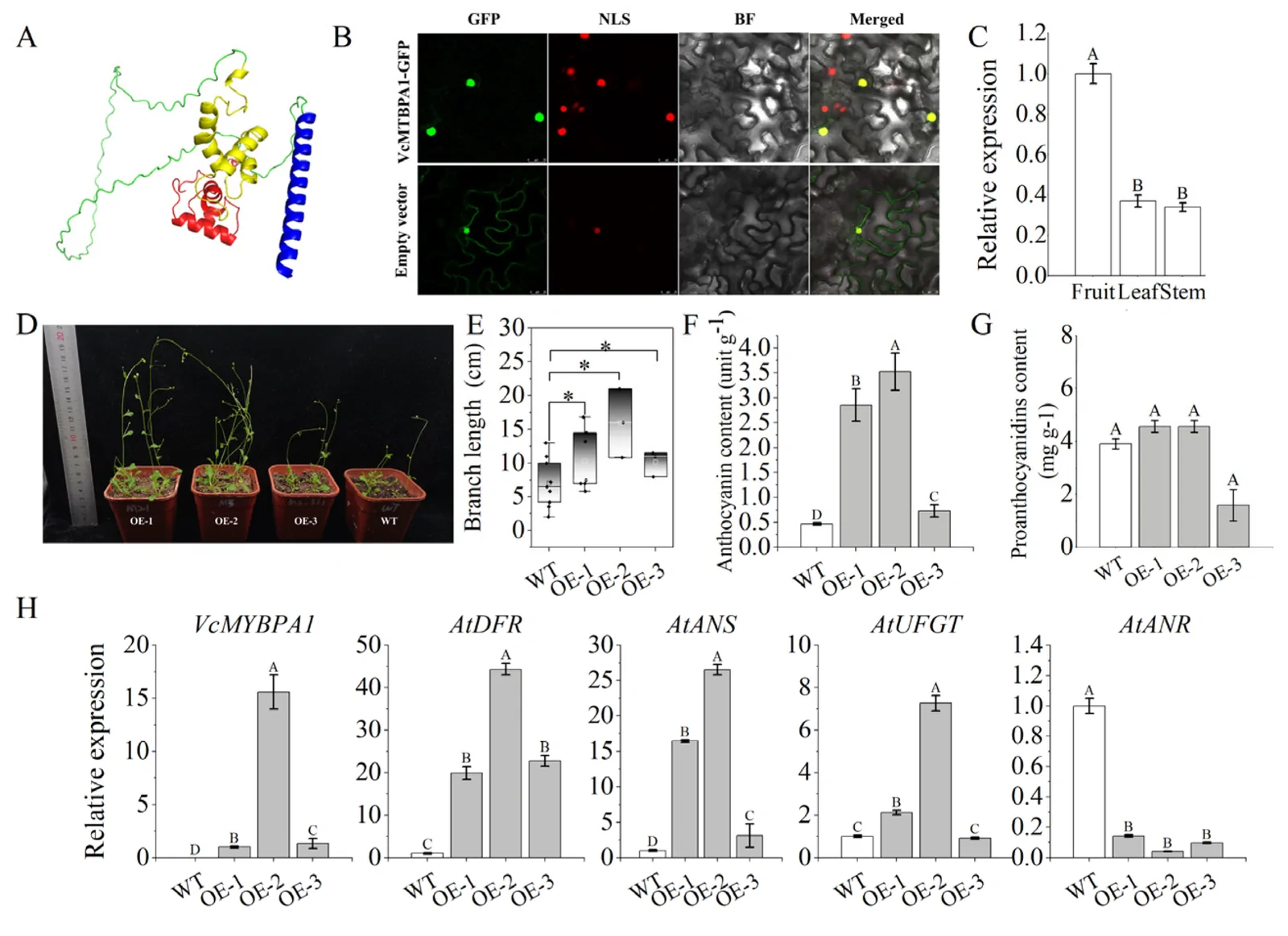
Functional analysis of *VcMYBPA1*. (**A**) represents protein tertiary structure prediction of VcMYBPA1. (**B**) Subcellular localization of *VcMYBPA1* in tobacco leaves stably expressing an NLS nuclear marker. The VcMYBPA1–GFP fusion construct or empty GFP vector was transiently expressed by *Agrobacterium*-mediated infiltration. GFP, green fluorescence; NLS, red nuclear marker fluorescence; BF, bright-field image; Merged, overlay of GFP and NLS signals. (**C**) represents the expression patterns of *VcMYBPA1* gene in different tissues. (**D**) represents the growth of stable T3 generation *Arabidopsis thaliana* (*35S:VcMYBPA1*) under 16 h of light/8 h of darkness, with wild-type Columbia *Arabidopsis* as a control. (**E**) represents the lengths of *Arabidopsis thaliana* after 30 days of growth. (**F**,**G**) represent anthocyanin and proanthocyanidin content in *Arabidopsis* leaves, respectively, sampled after 60 days of *Arabidopsis* development, when the seeds had matured. (**H**) represents the expression levels of *VcMYBPA1* and structural genes involved in anthocyanin and proanthocyanidin biosynthesis (*AtDFR*, *AtANS*, *AtUFGT*, *AtANR*) in *Arabidopsis*. Uppercase letters indicate the analysis of differences between strains (*p* ≤ 0.05 by ANOVA, Duncan’s test, * represents *p* ≤ 0.05).

Our previous research demonstrated that the expression of *VcMYBPA1* is regulated by abscisic acid (ABA), low temperature, and light conditions. Histochemical staining analysis of GUS in transgenic *Arabidopsis* revealed enhanced GUS expression activity following LED light and continuous light treatments [47]. To further investigate the function of this gene, we analyzed its structure and functionality. VcMYBPA1 encodes a protein containing a Myb-DNA-binding domain (Figure 7A). Subcellular localization studies indicated that *VcMYBPA1* was mainly localized in the nucleus (Figure 7B). Tissue specificity analysis revealed expression of *VcMYBPA1* in fruits, leaves, and stems, with the highest expression level observed in fruits (Figure 7C). PCR analysis confirmed the presence of the introduced *VcMYBPA1* gene in all three lines (Supplementary Figure S5), and qRT-PCR analysis further confirmed the expression of *VcMYBPA1* in each line (Figure 7H). Heterologous expression of the *VcMYBPA1* gene in *Arabidopsis* resulted in significant elongation of branch length (Figure 7D,E). Overexpression of *VcMYBPA1* also led to an increase in anthocyanin content in *Arabidopsis* (Figure 7F), while having no significant effect on proanthocyanidin content (Figure 7G). Additionally, the overexpression of *VcMYBPA1* significantly up-regulated the expression levels of *AtDFR*, *AtANS*, and *AtUFGT* (Figure 7H). Heterologous overexpression of *VcMYBPA1* significantly increased the expression levels of *AtDFR*, *AtANS*, and *AtUFGT* (Figure 7H). However, these expression changes do not distinguish between direct transcriptional regulation and indirect downstream effects of VcMYBPA1 overexpression.

## 4. Discussion

### 4.1. Supplemental Lighting Accelerates Blueberry Fruit Ripening and Improves Quality-Related Traits

With the growing popularity of plant cultivation in greenhouses, there has been increased emphasis on the critical role of light in enhancing plant yield and quality, as well as its wide-ranging applications in fruit development. The results of this study indicate that supplemental lighting can lead to greater transverse and longitudinal fruit diameters and facilitate earlier ripening of blueberry fruit. At present, the ripening of blueberries is mainly judged by their appearance. Their apparent ripening is mainly distinguished by the color change of the fruits. In the treatment group, the proportion of whole fruit surfaces presenting blue-purple color reached on 23 April was much higher than that of the control treatment. In this study, as the fruit developed, in the treatment group, its color (L value and C value) significantly decreased, while the contents of fructose and sucrose significantly increased, and the titratable acidity decreased. Furthermore, supplemental blue light at various frequencies promoted earlier ripening of tomato fruits by approximately 3–4 days, leading to a significant increase in the levels of lycopene, total phenolic compounds, total flavonoids, vitamin C, soluble sugars, and overall antioxidant activity of the tomatoes [28]. Similarly, strawberries exhibited accelerated ripening when cultivated under blue LED light, and the antioxidant activity of the blue LED-illuminated fruits was significantly higher [13]. These findings suggest that the increase in these constituents partially reflects the earlier ripening of the fruit when compared to the control, as fruit ripening occurs only after the accumulation of these components reaches a certain threshold, with supplemental light facilitating this process.

Instantaneous gas-exchange measurements conducted at the fruit color transition stage showed that supplemental lighting increased the leaf net photosynthetic rate, stomatal conductance, and intercellular CO_2_ concentration, but the transpiration rate was not significantly affected (Supplementary Figure S6). These results indicate improved instantaneous leaf gas-exchange performance at this developmental stage and are consistent with the higher soluble sugar contents observed in the fruit. However, because gas-exchange parameters were measured at only one time point and neither chlorophyll fluorescence nor photosynthate allocation was examined, the present data do not establish a direct causal relationship between enhanced photosynthetic carbon assimilation, sugar accumulation, and anthocyanin biosynthesis. Repeated physiological measurements and carbon-allocation analyses throughout fruit development will be required to clarify these relationships.

An additional limitation is that the natural light intensity, spectral composition, and daily light integral inside the greenhouse were not continuously monitored. Therefore, the observed effects cannot be attributed exclusively to photoperiod extension and likely resulted from the combined effects of a longer daily light period and increased light availability during the low-light winter season. Future studies should continuously monitor greenhouse light conditions and compare treatments with equivalent daily light integrals but different photoperiods to distinguish the effects of photoperiod from those of total photon exposure.

### 4.2. Supplemental Lighting Is Associated with Anthocyanin Biosynthesis, Transport, and Signaling Pathways

Blueberries are particularly rich in anthocyanins [20]. During the early stages of fruit development, proanthocyanidins and flavonols are the predominant phenolic compounds, while anthocyanins begin to accumulate during ripening [48]. This was confirmed in the present study, where anthocyanin content increased quickly after S4 (turning stage), whereas levels of PAs and flavonols decreased. Transcriptome analysis indicated that differentially up-regulated gene enrichment pathways were primarily associated with genes involved in galactose metabolism and anthocyanin biosynthesis. Galactoside synthase genes were differentially up-regulated, catalyzing the formation of UDP-galactose, a key glycosyl donor in anthocyanin glycosylation to form anthocyanidins. Although malvidin-3-galactoside has been identified as the major anthocyanin component in ‘O’Neal’ blueberry fruit [49], the present study only quantified total anthocyanin content via a spectrophotometric method without resolving individual anthocyanin monomers. Accordingly, the observed elevation in total anthocyanins cannot be specifically attributed to changes in delphinidin-, cyanidin-, petunidin-, peonidin-, or malvidin-derived glycosides.

Most genes in the phenylalanine and anthocyanin pathways, such as *CHS*, *CHI*, *F3H*, *F3′H*, *DFR*, and *ANS*, are responsive to light signaling [6,27,50,51]. Structural genes involved in the phenylpropanoid pathway were up-regulated following the supplementation of red and blue light during the ripening stages of lingonberries [6]. Our previous study showed that the genes *VcDFR*, *VcANS*, and *VcUFGT* are aligned with anthocyanin biosynthesis in response to transient exposure to UV-A, B, and C light [27]. In this study, we observed a significant positive correlation between the trend of anthocyanin content and the expression of genes *VcF3′5′H*, *VcANS*, *VcUFGT*, and *VcMYBA1*. Moreover, there was a positive correlation between anthocyanin content and the expression of *VcCHS*, *VcCHI*, *VcF3H,* and *VcDFR* under supplemental lighting treatments. Furthermore, at the fruit ripening stage (S4–S5), these genes were expressed at higher levels compared to the control. These findings suggest that flavonoid pathway-related genes (*VcCHS*, *VcCHI*, *VcF3′5′H*, and *VcF3H*) may play a more significant role in the anthocyanin synthesis pathway of blueberries in response to supplemental lighting. Across fruit development, anthocyanin content was positively correlated with sucrose, fructose, and glucose contents and was significantly correlated with the expression levels of *VcANS* and *VcUFGT*. Previous studies have shown that exogenous sucrose promotes anthocyanin accumulation in *Arabidopsis* [52]. In the present study, the coordinated changes in soluble sugar and anthocyanin contents suggest a potential association between carbohydrate status and anthocyanin accumulation. However, because photosynthetic carbon assimilation and sugar-signaling pathways were not directly examined, these results do not establish a causal relationship between sugar accumulation and anthocyanin biosynthesis. Further physiological and functional analyses are therefore required.

Furthermore, transcriptome analysis revealed that differentially up-regulated genes were enriched for functions related to transmembrane transport. Four classes of proteins, including glutathione S-transferase (GST), multidrug resistance-associated proteins (MRPs), multidrug and toxin extrusion (MATE) proteins, and bacteriophage-like transporters (BTLs), have been identified as potentially essential for the transport of anthocyanidin glycosides to vesicles [6]. Moreover, SNARE structural domain transporter proteins are candidates for transmembrane transport functions involved in transporting anthocyanidins either to vesicles or from the endoplasmic reticulum (ER) to the Golgi apparatus and endosomes [6]. Furthermore, in the KEGG enrichment analysis, the phosphatidylinositol pathway was enriched and up-regulated. The phosphatidylinositol pathway is one of the pathways in G-protein-coupled receptor signal transduction, and this signaling pathway is closely related to vesicle transport [53]. These results indicate that in addition to affecting the synthesis of anthocyanins, supplemental lighting treatment also affects the transport of anthocyanins.

GO and KEGG enrichment analyses showed that the genes up-regulated by supplemental lighting were significantly enriched in calcium ion transport, calmodulin binding, and phosphatidylinositol signaling, suggesting that these pathways may participate in the signaling response of blueberry fruit to supplemental lighting. However, enrichment analysis cannot determine the upstream or downstream positions of these pathways within a regulatory network. Therefore, they cannot be directly defined as upstream regulatory pathways of supplemental-light-induced anthocyanin accumulation. The exploratory pathway–gene–correlation network constructed in this study showed that several calcium transport- and calcium-responsive candidate genes, including *CML*, *CPK*, *MCU*, *ATP2C*, and *CBP60B*-related genes, exhibited significant expression covariation with light-signaling candidates and anthocyanin-associated genes, including *VcMYBPA1*, *Vc4CL*, and *VcUFGT*. Ca^2+^ can act as a second messenger in plant responses to environmental signals and may affect protein phosphorylation and transcriptional responses through components such as calmodulin/calmodulin-like proteins and calcium-dependent protein kinases. Recent studies have also indicated that light perception may be functionally connected with Ca^2+^/calmodulin-responsive transcriptional networks and that changes in Ca^2+^ availability can affect anthocyanin accumulation and the expression of related genes, although the direction of these effects depends on the species and treatment conditions [54,55]. Therefore, the observed expression associations between calcium-related candidates and anthocyanin-associated genes provide preliminary evidence of a possible functional connection but do not demonstrate direct regulation. The network also revealed significant expression associations of phosphatidylinositol-signaling candidates, including *PLCD*, *PIP5K*, and *dgkA*-related genes, with *VcMYBPA1*, *Vc4CL*, *VcUFGT*, and several light-signaling candidates. Phosphoinositides participate in membrane-associated signaling and vesicle trafficking, and the dynamics of PI(4,5)P_2_ can affect the localization and movement of secretory vesicles at the plasma membrane [56]. On this basis, we hypothesize that supplemental lighting may be accompanied by changes in calcium transport- and phosphatidylinositol signaling-related processes, which may functionally converge with MYB-associated transcriptional responses and anthocyanin biosynthesis. However, this model remains a hypothesis based on pathway enrichment and expression covariation. Further measurements of cytosolic Ca^2+^ dynamics, phosphoinositide abundance, protein phosphorylation, and candidate–gene interactions will be required to validate these proposed relationships.

### 4.3. Functional Characterization of VcMYBPA1 and Its Association with Anthocyanin Accumulation

MYBPA1-type transcription factors have traditionally been regarded as key regulators of proanthocyanidin biosynthesis in *Vaccinium* species [57]. However, increasing evidence indicates that *MYBPA1* proteins also contribute to anthocyanin accumulation. In bilberry (*Vaccinium myrtillus*), silencing of *VmMYBPA1.1* significantly reduced the expression of flavonoid biosynthetic genes and anthocyanin accumulation, while promoter activation assays demonstrated that *VmMYBPA1.1* directly activates several anthocyanin biosynthetic genes, including *F3′5′H*, *DFR*, *ANS*, and *UFGT* [45]. Similar functions have been reported in other *Vaccinium* species, suggesting that *MYBPA1* may play a broader role in flavonoid metabolism beyond proanthocyanidin biosynthesis [58,59].

In the present study, supplemental lighting significantly increased both anthocyanin accumulation and *VcMYBPA1* expression during the ripening stage of blueberry fruit. Consistent with these observations, heterologous overexpression of *VcMYBPA1* in *Arabidopsis* increased anthocyanin accumulation. This result supports a role for *VcMYBPA1* in anthocyanin-associated processes but does not establish its direct transcriptional targets in blueberry. In addition to enhanced anthocyanin accumulation, all three independent *VcMYBPA1*-overexpressing lines exhibited increased branch length. To date, MYBPA-type transcription factors have been primarily associated with the regulation of proanthocyanidin and anthocyanin metabolism, and direct evidence linking MYBPA proteins to branch elongation remains limited. For example, ectopic expression of *VvMYBPA1* induced proanthocyanidin accumulation in several vegetative tissues of *Arabidopsis* [60], whereas overexpression of the MYBPA1-type gene *PtMYB115* in poplar did not produce detectable growth or developmental abnormalities [61]. Therefore, the branch-elongation phenotype observed here may represent a species- or expression-context-dependent pleiotropic effect of heterologous *VcMYBPA1* expression. Its relationship with anthocyanin accumulation cannot yet be determined and requires further investigation of internode development, hormone signaling, carbon allocation, and biomass accumulation.

The relationship between MYBPA1 and *UFGT* remains controversial in *Vaccinium*. Karppinen [45] reported that VmMYBPA1.1 activated the *VmUFGT* promoter in bilberry, whereas Lafferty [48] found that VcMYBPA1.1 failed to activate the *VcUFGT* promoter in a tobacco transient-expression system. In the present study, *VcMYBPA1* expression was positively correlated with *VcUFGT* expression under supplemental lighting, and *AtUFGT* expression was higher in VcMYBPA1-overexpressing *Arabidopsis* than in the wild type. These observations indicate an expression association but do not establish regulatory direction or causality. The correlation may reflect a shared response to supplemental lighting or fruit development, while the increase in *AtUFGT* expression may represent either a direct effect or an indirect downstream response.

Sequence analysis identified several putative MYB-binding motifs in the *VcUFGT* promoter, including CAACTG, TAACCA, and CAACCA (Supplementary Table S6). However, the presence of predicted cis-elements alone does not demonstrate functional binding or transcriptional activation. Previous identification of light-responsive elements in the *VcMYBPA1* promoter [48] further suggests that *VcMYBPA1* may be associated with the transcriptional response to supplemental lighting, but the current evidence does not establish a direct light–VcMYBPA1–VcUFGT regulatory pathway. Moreover, because MYB proteins commonly function together with bHLH and WDR partners [44], the composition and activity of a VcMYBPA1-associated MBW complex remain unresolved. Direct promoter regulation and MBW-complex formation will require validation using promoter activity, DNA-binding, and protein interaction assays.

## 5. Conclusions

Supplemental lighting promoted blueberry fruit enlargement, accelerated fruit ripening, and improved several quality-related traits, including soluble sugar, vitamin C, and flavonoid accumulation. Transcriptome and qRT-PCR analyses showed that supplemental lighting affected genes involved in galactose metabolism, anthocyanin biosynthesis, and *MYB* transcriptional regulation. Among these genes, *VcMYBPA1* showed a close expression association with *VcUFGT* and anthocyanin accumulation, whereas heterologous overexpression of *VcMYBPA1* in *Arabidopsis* enhanced anthocyanin accumulation and was accompanied by increased expression of anthocyanin-related structural genes. Overall, this study provides useful physiological and molecular information for improving blueberry fruit quality through supplemental lighting in greenhouse cultivation.

## Data Availability

The data presented in this study are available on request from the corresponding author.

## Supplementary Materials

The following supporting information can be downloaded at: https://www.mdpi.com/article/10.3390/plants15172671/s1, Figure S1: Yield per tree. Three trees were randomly selected for each treatment and yield per tree was calculated by multiplying the average fruit weight by the number of fruits per tree. Lowercase letters represent the analysis of differences among different treatments within the same period (using *t*-test, *p*-value ≤ 0.05); Figure S2: The correlation analysis of gene expression levels between the treatment group and the control group samples. The correlation algorithm used was Pearson, with complete clustering and Euclidean distance calculation method; Figure S3: Target-centered exploratory correlation network linking anthocyanin-associated genes with candidate genes involved in calcium signaling, phosphatidylinositol signaling, and light signaling. Different colors represent different functional modules, and red solid lines indicate positive correlations; Figure S4: The evolutionary tree analysis. VcMYB9 (ALP43796.1, Vaccinium corymbosum), VmMYBPA1.1 (QWW89542.1, *Vaccinium myrtillus*), VuMYBPA1.2 (AKC94840.1, *Vaccinium uliginosum*), VvMYBPA1 (NP_001268160.1, *Vitis vivax*), VvMYBPA1 (NP_001268160.1, *Vitis vivax*), VmMYBPA1.2 (QWW89543.1, *Vaccinium myrtillus*), VvMYBPA1 (NP_001268160.1, *Vitis vinifera*), VvMYB4 (RVW17057.1, *Vitis vinifera*), VmMYBC2.1 (QWW89538.1, *Vaccinium myrtillus*), VcMYB21 (ALP43795.1, *Vaccinium corymbosum*), VcMYB1 (ALP43799.1, *Vaccinium corymbosum*), VcMYBA1 (AYE53992.1, *Vaccinium corymbosum*), MYB36 (OAO95809.1, *Arabidopsis thaliana*), PaMYB14 (XP_034931777.1, *Populus alba*), VcMYB14 (URH24710.1, *Vaccinium corymbosum*), RvMYB62 (XP_058227506.1, *Rhododendron vialii*), VvMYB108 (WES00146.1, *Vitis vinifera*), VvMYB62 XP_002276526.3, *Vitis vinifera*), AtMYB73 (NP_195443.1, *Arabidopsis thaliana*). Phylogenetic analysis was conducted using MEGA v11.0, with bootstrap values calculated via the neighbor—joining method over 1000 replicates; Figure S5: PCR verification of the VcMYBPA1-overexpressing lines; Figure S6: Effects of early-morning supplemental lighting on instantaneous leaf gas-exchange parameters of greenhouse-grown blueberry plants at the turning stage. (A) Net photosynthetic rate (Pn); (B) stomatal conductance (Gs); (C) intercellular CO_2_ concentration (Ci); and (D) transpiration rate (Tr). Values are presented as the means ± SE (*n* = 10). Different lowercase letters indicate significant differences between the supplemental-lighting treatment and the control at *p* < 0.05 according to *t*-test; Figure S7: Analysis of the interaction between the transcription factor VcMYBPA1 and DNA (MYB binding site sequences). Predictions were made using AlphaFold3 and visualized with PyMOL, where chains B–F correspond to the MYB binding elements (CAACTG, TAACCA, CAACCA, CAACAG, CCGTTG). The blue cartoon representation depicts the VcMYBPA1 protein, while the yellow sticks represent DNA. Red indicates atoms within 5.0 Å of any atom in the VcMYBPA1 protein, and orange denotes atoms in the DNA that are within 5.0 Å of any atom in the VcMYBPA1 protein; Table S1: Primer names and sequences; Table S2: Statistical table of sequencing data; Table S3: Statistical table of comparison results; Table S4: Strong expression correlations between anthocyanin-associated target genes and candidate genes involved in calcium, phosphatidylinositol, and light signaling; Table S5: Annotation-based screening and expression profiles of bHLH, and WDR candidate; Table S6: Cis-acting elements of the VcUFGT promoter.
