# Supplementary material for: Supplemental Lighting Enhances Blueberry Fruit Quality and Flavonoid Accumulation Associated with VcMYBPA1 Expression in Greenhouses
Source: Plants (Basel). 2026 Aug 31;15(17):2671. doi: 10.3390/plants15172671 (PMC13567242; doi:10.3390/plants15172671)
Supplement: Supplementary file 1 [file plants-15-02671-s001.zip › plants-4458553-supplementary.pdf]

Supplementary Figures

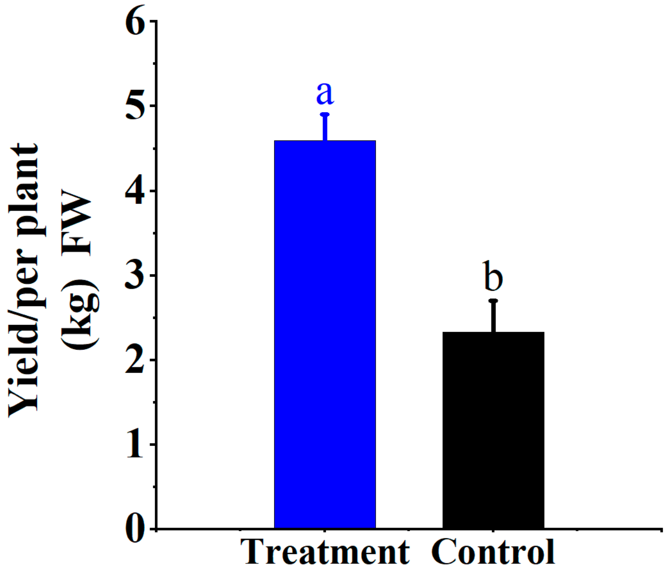

Supplementary Figure S1. Yield per tree. Three trees were randomly selected for each treatment and yield per tree was calculated by multiplying the average fruit weight by the number of fruits per tree. Lowercase letters represent the analysis of differences among different treatments within the same period (using T-test, P-value ≤ 0.05).

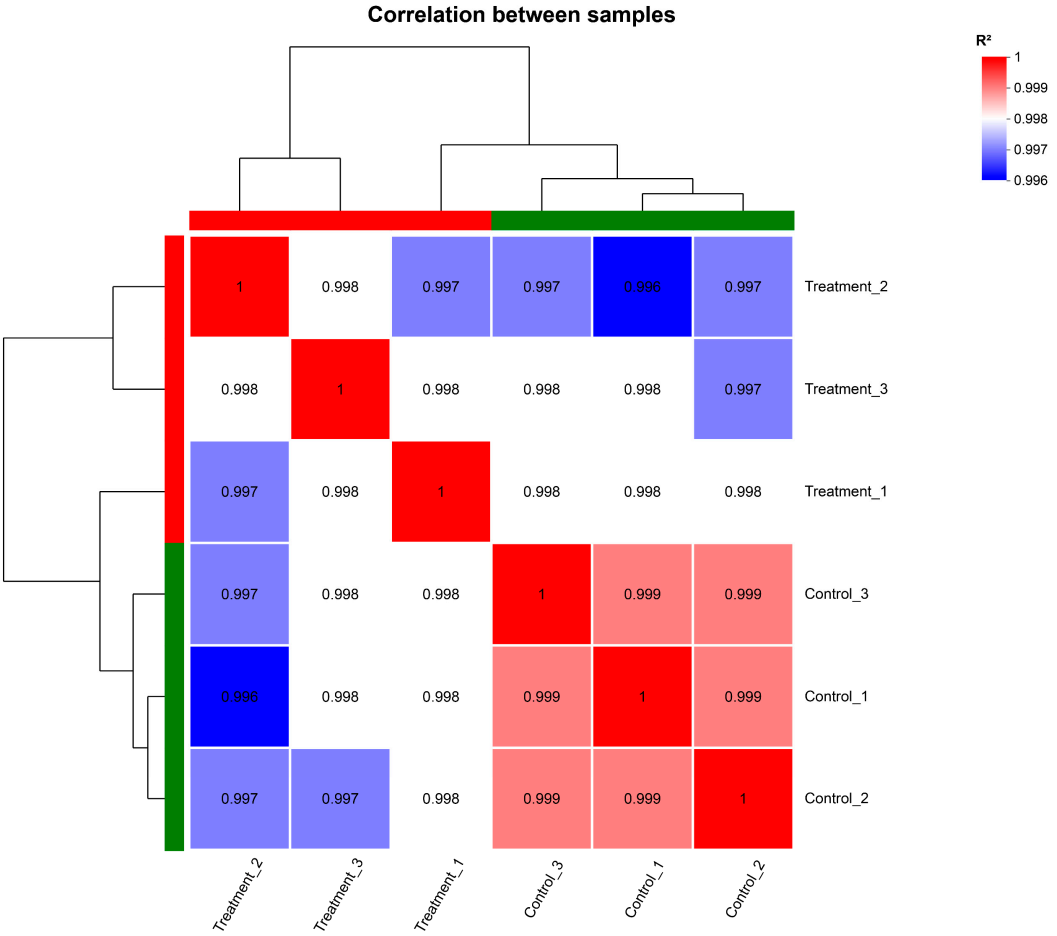

Supplementary Figure S2. The correlation analysis of gene expression levels between the treatment group and the control group samples. The correlation algorithm used was Pearson, with complete clustering and Euclidean distance calculation method.

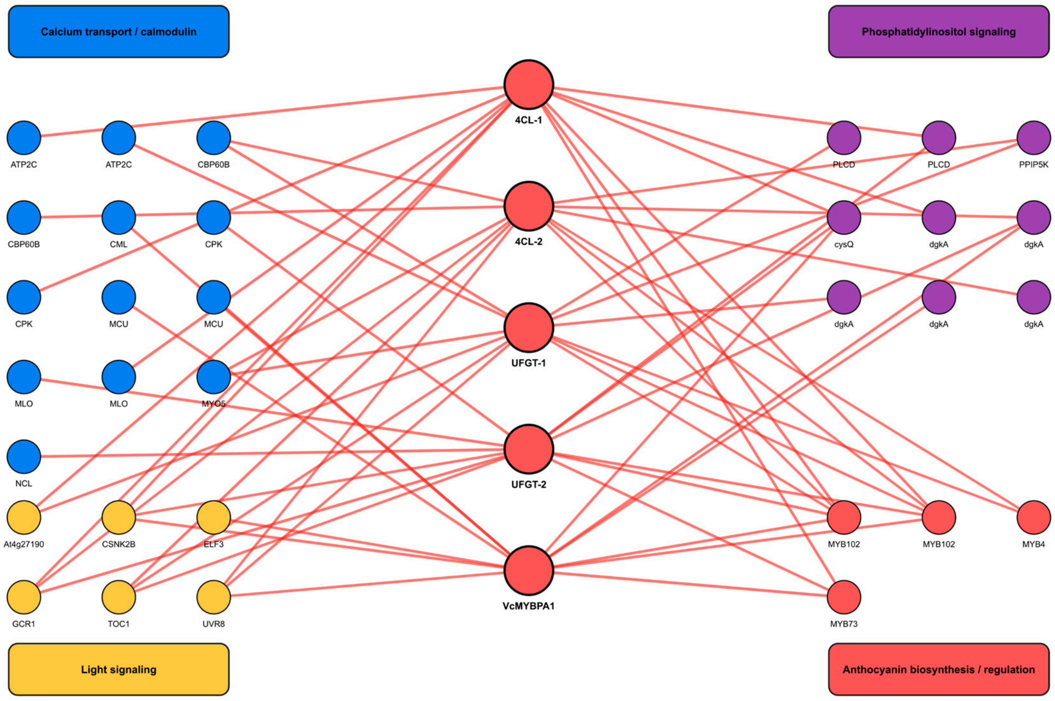

Supplementary Figure S3. Target-centered exploratory correlation network linking anthocyanin-associated genes with candidate genes involved in calcium signaling, phosphatidylinositol signaling, and light signaling. Different colors represent different functional modules, and red solid lines indicate positive correlations.

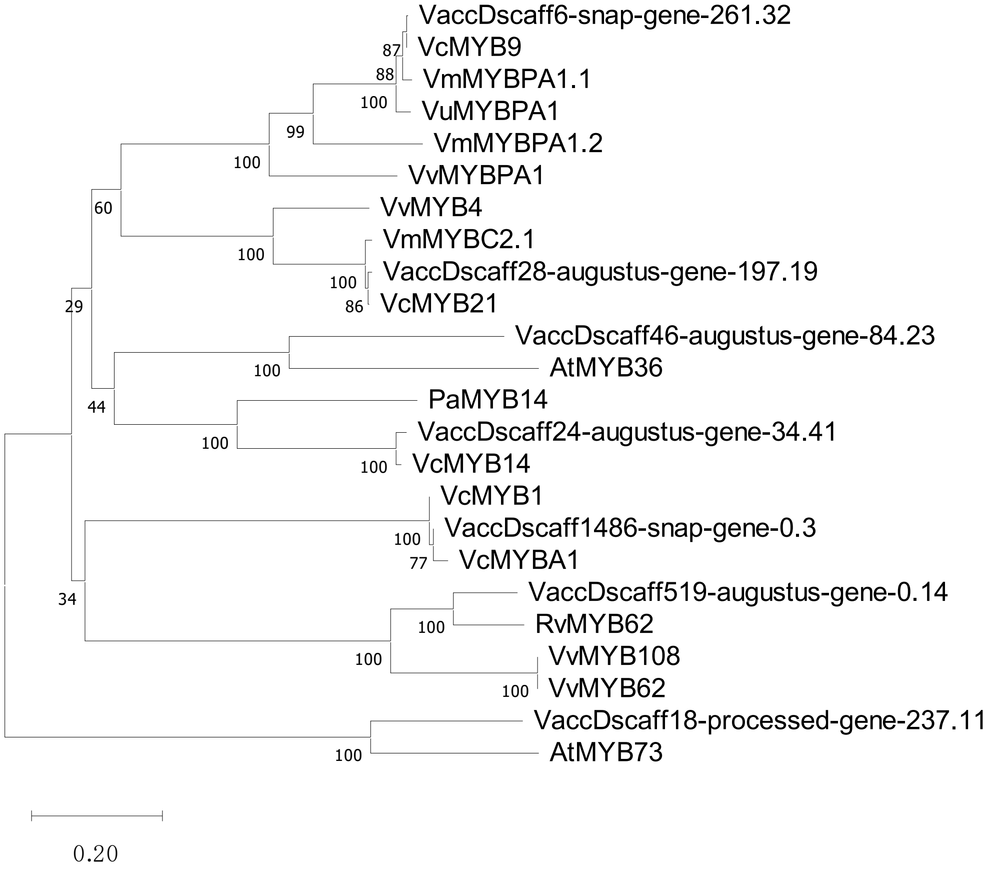

Supplementary Figure S4. The evolutionary tree analysis. VcMYB9 (ALP43796.1, *Vaccinium corymbosum*), VmMYBPA1.1 (QWW89542.1, *Vaccinium myrtillus*), VuMYBPA1.2 (AKC94840.1, *Vaccinium uliginosum*), VvMYBPA1 (NP_001268160.1, *Vitis vivax*), VvMYBPA1 (NP_001268160.1, *Vitis vivax*), VmMYBPA1.2 (QWW89543.1, *Vaccinium myrtillus*), VvMYBPA1 (NP_001268160.1, *Vitis vinifera*), VvMYB4 (RVW17057.1, *Vitis vinifera*), VmMYBC2.1 (QWW89538.1 , *Vaccinium myrtillus*), VcMYB21 (ALP43795.1 , *Vaccinium corymbosum*), VcMYB1 (ALP43799.1 , *Vaccinium corymbosum*), VcMYBA1 (AYE53992.1, *Vaccinium corymbosum*), MYB36 (OAO95809.1, *Arabidopsis thaliana*), PaMYB14 (XP_034931777.1 , *Populus alba*), VcMYB14 (URH24710.1, *Vaccinium corymbosum*), RvMYB62 (XP_058227506.1 ,*Rhododendron vialii*), VvMYB108 (WES00146.1, *Vitis vinifera*), VvMYB62 XP_002276526.3, *Vitis vinifera*), AtMYB73 (NP_195443.1, *Arabidopsis thaliana*). Phylogenetic analysis was conducted using MEGA v11.0, with bootstrap values calculated via the neighbor–joining method over 1000 replicates.

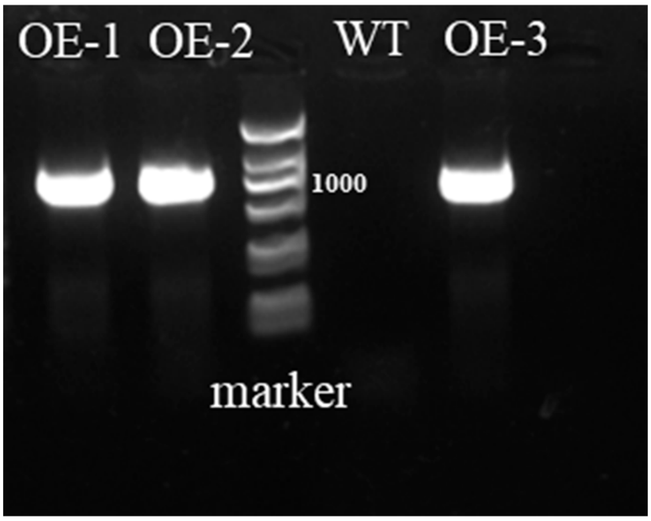

Supplementary Figure S5. PCR verification of the VcMYBPA1-overexpressing lines.

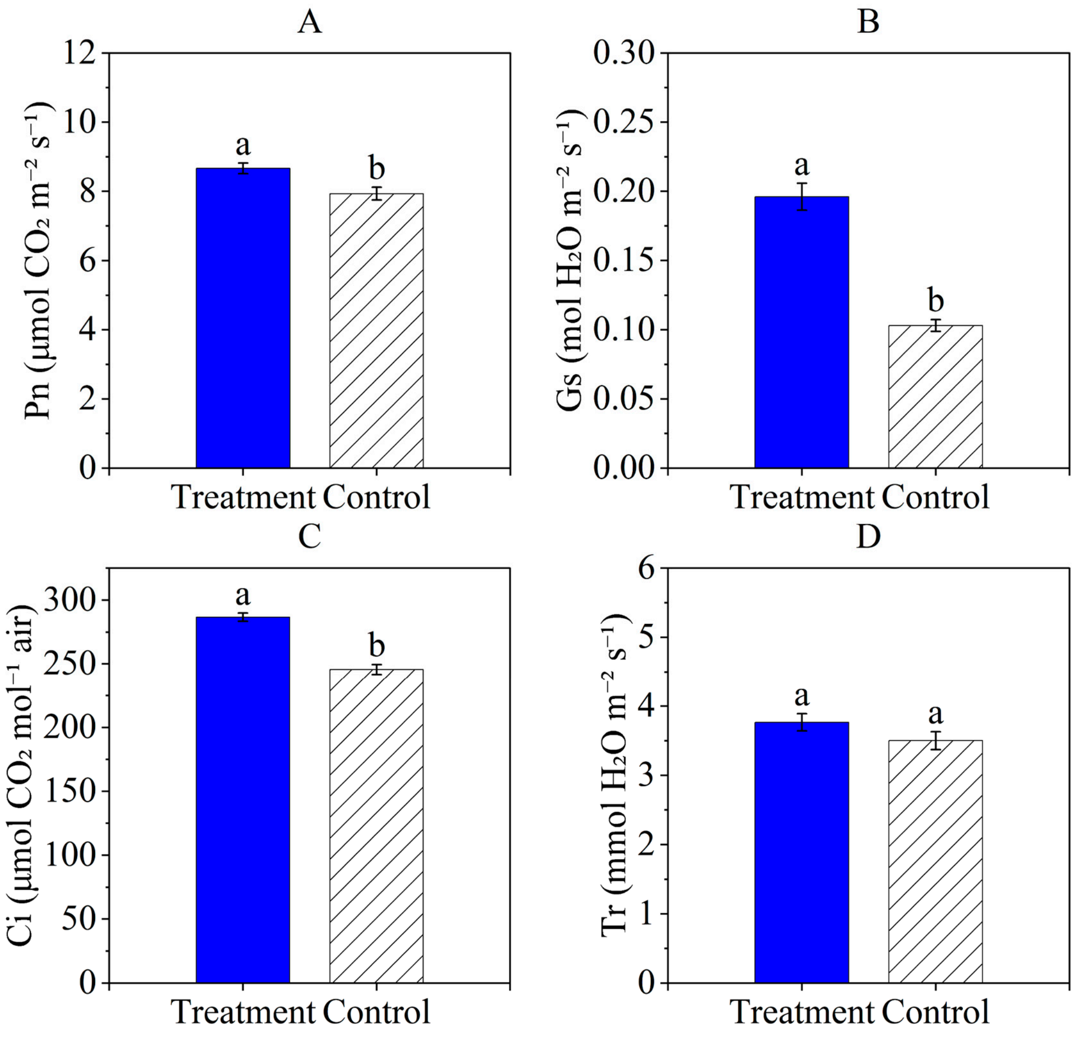

Supplementary Figure S6. Effects of early-morning supplemental lighting on instantaneous leaf gas-exchange parameters of greenhouse-grown blueberry plants at the turning stage. (A) Net photosynthetic rate (Pn); (B) stomatal conductance (Gs); (C) intercellular CO_2 concentration (Ci); and (D) transpiration rate (Tr). Values are presented as the means ± SE (n =10). Different lowercase letters indicate significant differences between the supplemental-lighting treatment and the control at $P < 0.05$ according to t-test.

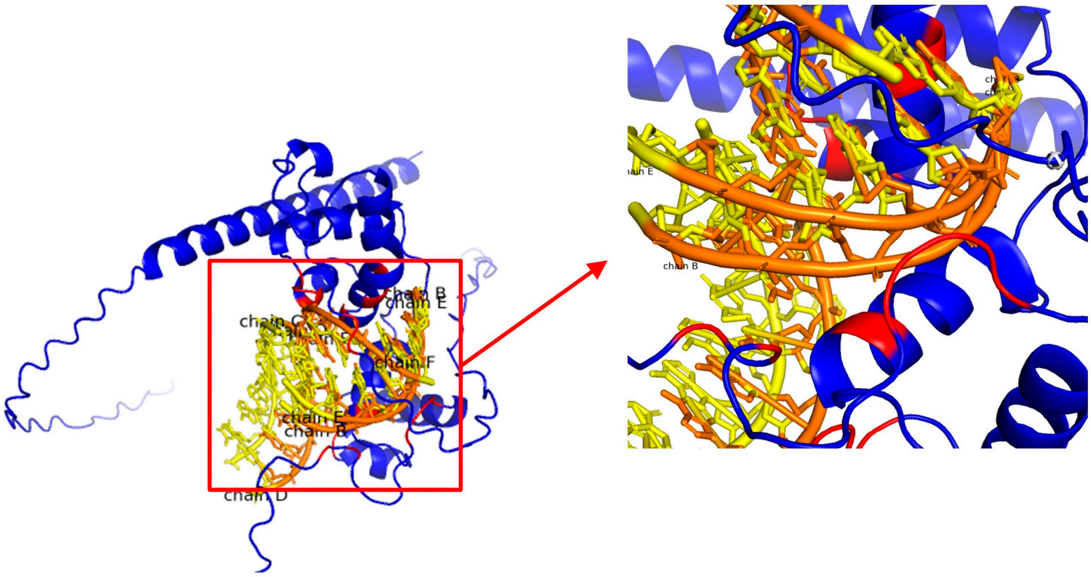

Supplementary Figure S7. Analysis of the interaction between the transcription factor VcMYBPA1 and DNA (MYB binding site sequences). Predictions were made using AlphaFold3 and visualized with PyMOL, where chains B-F correspond to the MYB binding elements (CAACTG, TAACCA, CAACCA, CAACAG, CCGTTG). The blue cartoon representation depicts the VcMYBPA1 protein, while the yellow sticks represent DNA. Red indicates atoms within 5.0 Å of any atom in the VcMYBPA1 protein, and orange denotes atoms in the DNA that are within 5.0 Å of any atom in the VcMYBPA1 protein.

Supplementary Tables

Supplementary Table S1. Primer names and sequences

Gene	Forward primer (5'-3')	Reverse primer (5'-3')
VcGAPDH-qRT-PCR	GGTTATCAATGATAGGTTTGGCA	CAGTCCTTGCTTGATGGAC
VcUBC28-qRT-PCR	*CAGTGCTGGTCCTGTTGGTGAAG*	*TGGCGGCTTGAATGGATAATCTGG*
VcPAL-qRT-PCR	GCAAACGGTGAGAGTGAGAGGAATC	CCTGTTTCCAATACCTGCCATCCC
VcLAR-qRT-PCR	TGATGAAGTTGAAGTGTGCGA	AATTCCATTGGCGGTTACG
VcFLS-qRT-PCR	GGGATACCGGAGGAAGTGAT	AGAAGGAGGCCAAACCTTGT
VcUFGT-qRT-PCR	GCGTATATCGGCTTCGGATCAGTG	AGAGACCAGAGGAACGGAGTGC
VcANS-qRT-PCR	CTGCAACTCTTCTACGAGGG	CCATTGCTCAAAATCTCGACC
VcANR-qRT-PCR	CAAAGAGGGATTCAGCTACAAGTA	ACAGACACACAGTGGCACATTAG
VcMYBA1-qRT-PCR	TCCCAAACACAACCACCGAG	CATTTGATCCGTCATTGGTC
VcDFR-qRT-PCR	CCGATACAAACCTAACGCTGTG	CTCAAAATCCATAGGTGTGGC
VcF3'5'H-qRT-PCR	ATAGGGCAAGTCATACTCAAGC	AAATAGTCCAGCCGAGGTCA
VcCHS-qRT-PCR	CGCATGTGTGACAAATCCCA	CATATCCTGCCTAGCGTCCA
VcCHI-qRT-PCR	CGAGAATGCGAATGTGGTTGAGATG	GCTAAGCAGGAGATGGTTGAAGGAG
VcC4H-qRT-PCR	TTGCACATTTTGAAGCACTCC	CCATTTCCTCCCCAACCCAA
Vc4CL-qRT-PCR	TGTATTTCCACAGCGAGGAC	CCACATAGCATTACCGAGT
VcF3H-qRT-PCR	AAGCTCCGGTTCGATATGTCC	ACTATTTCTCTCCAGTCTTGCAC
VcF3'H-qRT-PCR	TTCACAGCAGGAACTGACACC	AGGCCCAACAACTGAGTCC
VcMYBPA1-qRT-PCR	ATTCAACTTCATGGCGAAGG	TCCCGCAATTAGAGACCATC
AtANS-qRT-PCR	AGTGATTACATAGAAGCAACGAGTGAG	ACCGACAGAGAGAGCCTTGAAG
AtDFR-qRT-PCR	TCTATGCCGCCTAGCCTTATCAC	TGTCCTTGTCTTATGATCGAGTAATGC
AtANR-qRT-PCR	AGAGCTTGGCGACCTGAAGATC	CGGAGTTGCGACATGGAAGATG
AtUFGT-qRT-PCR	CTCGCCGCCGCTTCTCC	CGTCGTGGACCTTGATGTTCTC
Atactin-qRT-PCR	GGAGCTGAGAGATTCCGTTG	GGTGCAACCACCTTGATCTT
maker-VaccDscaff6-snap-gene-261.32	acgggggactcttgaccatggATGGATGTGGATCCAAACACTTT	tactagtcagatctaccatggAATCAACAATGATTCGGCAAAGG

Supplementary Table S2. Statistical table of sequencing data

Sample	Raw reads	Raw bases	Clean reads	Clean bases	Error rate(%)	Q20(%)	Q30(%)	GC content(%)
Control_1	49030458	7403599158	48531798	7229178197	0.0122	98.62	95.63	46.55
Control_2	47168702	7122474002	46746774	6970304109	0.0122	98.63	95.64	46.53
Control_3	49998810	7549820310	49540252	7377450499	0.0122	98.65	95.68	46.5
Treatment_1	52233542	7887264842	51741672	7704645584	0.0122	98.63	95.65	46.56
Treatment_2	44605258	6735393958	44274186	6579191808	0.0121	98.7	95.86	46.08
Treatment_3	54520896	8232655296	54103080	8051035737	0.0121	98.7	95.83	46.07

Supplementary Table S3. Statistical table of comparison results

Sample	Total reads	Total mapped	Multiple mapped	Uniquely mapped
Control_1	48531798	45190002(93.11%)	15881838(32.72%)	29308164(60.39%)
Control_2	46746774	43525442(93.11%)	15232594(32.59%)	28292848(60.52%)
Control_3	49540252	46092996(93.04%)	16263400(32.83%)	29829596(60.21%)
Treatment_1	51741672	48169210(93.1%)	16961669(32.78%)	31207541(60.31%)
Treatment_2	44274186	41174572(93.0%)	14934705(33.73%)	26239867(59.27%)
Treatment_3	54103080	50293864(92.96%)	18033251(33.33%)	32260613(59.63%)

Supplementary Table S4. Strong expression correlations between anthocyanin-associated target genes and candidate genes involved in calcium, phosphatidylinositol, and light signaling.

Gene 1	Symbol 1	Gene 2	Symbol 2	Pearson r	Pearson P	Spearman rho	Spearman P	Pearson FDR	Spearman FDR
maker-VaccDscaff1-augustus-gene-350.36	ATP2C	maker-VaccDscaff19-snap-gene-64.36	UFGT-1	0.9959264	2.486E-05	0.9428571	0.0048047	0.0072434	0.0355952
maker-VaccDscaff19-snap-gene-64.36	UFGT-1	maker-VaccDscaff47-augustus-gene-152.22	MYO5	0.9890932	0.0001778	1	0	0.0084202	0
maker-VaccDscaff19-snap-gene-64.36	UFGT-1	maker-VaccDscaff25-augustus-gene-325.43	CBP60B	0.9874343	0.0002359	1	0	0.0088279	0
maker-VaccDscaff16-snap-gene-49.29	PPIP5K	maker-VaccDscaff19-snap-gene-64.36	UFGT-1	0.950329	0.0036395	0.7714286	0.0723965	0.0152398	0.1204587
maker-VaccDscaff19-snap-gene-64.36	UFGT-1	maker-VaccDscaff39-augustus-gene-139.18	dgkA	0.9497566	0.0037232	0.8857143	0.0188455	0.0153441	0.0777843
maker-VaccDscaff19-snap-gene-64.36	UFGT-1	maker-VaccDscaff20-augustus-gene-41.51	PLCD	0.9301357	0.007151	0.8857143	0.0188455	0.0195973	0.0777843
maker-VaccDscaff19-snap-gene-64.36	UFGT-1	maker-VaccDscaff27-snap-gene-56.38	At4g27190	0.9401611	0.0052639	0.8285714	0.0415627	0.0174175	0.1163063
maker-VaccDscaff19-snap-gene-64.36	UFGT-1	maker-VaccDscaff4-snap-gene-355.37	TOC1	0.9204844	0.0092327	0.8857143	0.0188455	0.0217202	0.0777843
maker-VaccDscaff19-snap-gene-64.36	UFGT-1	maker-VaccDscaff28-snap-gene-111.33	UVR8	0.9081	0.0122803	0.7714286	0.0723965	0.0247589	0.1204587
maker-VaccDscaff19-snap-gene-64.36	UFGT-1	maker-VaccDscaff24-snap-gene-213.30	MYB102	0.9001819	0.0144482	0.7714286	0.0723965	0.0266469	0.1204587
maker-VaccDscaff19-snap-gene-64.36	UFGT-1	maker-VaccDscaff24-augustus-gene-34.41	MYB4	0.8658179	0.0257993	0.7142857	0.1107872	0.0371683	0.149414
maker-VaccDscaff15-augustus-gene-160.24	MYB102	maker-VaccDscaff19-snap-gene-64.36	UFGT-1	0.8488446	0.0325451	0.6	0.208	0.0434351	0.2176084
snap_masked-VaccDscaff7-processed-gene-315.23	CML	maker-VaccDscaff6-snap-gene-261.32	VcMYBPA1	0.976372	0.0008308	1	0	0.0103607	0

maker-VaccDscaff43-augustus-gene-72.24	MCU	maker-VaccDscaff6-snap-gene-261.32	VcMYBPA1	0.9588809	0.0025014	1	0	0.01335	0
maker-VaccDscaff22-augustus-gene-304.34	MCU	maker-VaccDscaff6-snap-gene-261.32	VcMYBPA1	0.9478108	0.0040145	0.9428571	0.0048047	0.0158377	0.0355952
maker-VaccDscaff38-augustus-gene-133.15	dgkA	maker-VaccDscaff6-snap-gene-261.32	VcMYBPA1	0.9336938	0.006449	0.9428571	0.0048047	0.0188223	0.0355952
maker-VaccDscaff37-augustus-gene-175.24	dgkA	maker-VaccDscaff6-snap-gene-261.32	VcMYBPA1	0.9040304	0.0133733	0.9428571	0.0048047	0.0257002	0.0355952
maker-VaccDscaff15-snap-gene-150.25	cysQ	maker-VaccDscaff6-snap-gene-261.32	VcMYBPA1	0.8986935	0.0148747	0.8285714	0.0415627	0.0271431	0.1163063
maker-VaccDscaff14-snap-gene-66.37	CSNK2B	maker-VaccDscaff6-snap-gene-261.32	VcMYBPA1	0.9572171	0.0027064	0.9428571	0.0048047	0.0137236	0.0355952
maker-VaccDscaff9-snap-gene-397.33	ELF3	maker-VaccDscaff6-snap-gene-261.32	VcMYBPA1	0.9207824	0.0091646	0.9428571	0.0048047	0.0216065	0.0355952
maker-VaccDscaff28-snap-gene-111.33	UVR8	maker-VaccDscaff6-snap-gene-261.32	VcMYBPA1	0.8935069	0.0164073	0.9428571	0.0048047	0.0285381	0.0355952
snap_masked-VaccDscaff18-processed-gene-237.11	MYB73	maker-VaccDscaff6-snap-gene-261.32	VcMYBPA1	0.9820128	0.0004824	1	0	0.0097103	0
maker-VaccDscaff15-augustus-gene-160.24	MYB102	maker-VaccDscaff6-snap-gene-261.32	VcMYBPA1	0.9265897	0.0078858	0.9428571	0.0048047	0.0202758	0.0355952
maker-VaccDscaff24-snap-gene-213.30	MYB102	maker-VaccDscaff6-snap-gene-261.32	VcMYBPA1	0.9242938	0.0083802	0.9428571	0.0048047	0.0207165	0.0355952
maker-VaccDscaff238-augustus-gene-0.55	MLO	maker-VaccDscaff38-augustus-gene-9.29	UFGT-2	0.9780078	0.0007202	0.840668	0.0360576	0.010068	0.1163063
maker-VaccDscaff38-augustus-gene-9.29	UFGT-2	maker-VaccDscaff41-augustus-gene-274.23	CPK	0.9714609	0.0012101	0.7714286	0.0723965	0.0111023	0.1204587
maker-VaccDscaff38-augustus-gene-9.29	UFGT-2	maker-VaccDscaff5-augustus-gene-393.32	NCL	0.9704194	0.0012996	0.9428571	0.0048047	0.0113387	0.0355952
maker-VaccDscaff38-augustus-gene-133.15	dgkA	maker-VaccDscaff38-augustus-gene-9.29	UFGT-2	0.9667638	0.0016386	1	0	0.012073	0
maker-VaccDscaff19-augustus-gene-363.25	PLCD	maker-VaccDscaff38-augustus-gene-9.29	UFGT-2	0.9630561	0.0020221	0.7142857	0.1107872	0.0125899	0.149414

maker-VaccDscaff15-snap-gene-150.25	cysQ	maker-VaccDscaff38-augustus-gene-9.29	UFGT-2	0.9383732	0.0055798	0.7714286	0.0723965	0.0177413	0.1204587
maker-VaccDscaff11-snap-gene-59.16	GCR1	maker-VaccDscaff38-augustus-gene-9.29	UFGT-2	0.9806908	0.0005557	0.7714286	0.0723965	0.0097103	0.1204587
maker-VaccDscaff38-augustus-gene-9.29	UFGT-2	maker-VaccDscaff4-snap-gene-355.37	TOC1	0.9082187	0.0122491	0.9428571	0.0048047	0.0247416	0.0355952
maker-VaccDscaff14-snap-gene-66.37	CSNK2B	maker-VaccDscaff38-augustus-gene-9.29	UFGT-2	0.9009845	0.0142207	0.8857143	0.0188455	0.0264056	0.0777843
maker-VaccDscaff15-augustus-gene-160.24	MYB102	maker-VaccDscaff38-augustus-gene-9.29	UFGT-2	0.9666165	0.0016531	0.8857143	0.0188455	0.012073	0.0777843
maker-VaccDscaff24-snap-gene-213.30	MYB102	maker-VaccDscaff38-augustus-gene-9.29	UFGT-2	0.938231	0.0056053	0.8857143	0.0188455	0.0177413	0.0777843
maker-VaccDscaff38-augustus-gene-9.29	UFGT-2	snap_masked-VaccDscaff18-processed-gene-237.11	MYB73	0.933937	0.0064023	0.9428571	0.0048047	0.0188195	0.0355952
maker-VaccDscaff24-snap-gene-242.23	MLO	maker-VaccDscaff37-augustus-gene-303.53	4CL-1	0.9972987	1.094E-05	1	0	0.0069423	0
maker-VaccDscaff37-augustus-gene-303.53	4CL-1	maker-VaccDscaff42-augustus-gene-20.28	CPK	0.9905482	0.0001336	1	0	0.0084202	0
maker-VaccDscaff37-augustus-gene-303.53	4CL-1	maker-VaccDscaff4-augustus-gene-57.34	ATP2C	0.9895143	0.0001643	0.9428571	0.0048047	0.0084202	0.0355952
maker-VaccDscaff19-augustus-gene-363.25	PLCD	maker-VaccDscaff37-augustus-gene-303.53	4CL-1	0.9806466	0.0005582	0.8857143	0.0188455	0.0097103	0.0777843
augustus_masked-VaccDscaff35-processed-gene-260.3	dgkA	maker-VaccDscaff37-augustus-gene-303.53	4CL-1	0.9525835	0.0033192	0.8857143	0.0188455	0.0148649	0.0777843
maker-VaccDscaff15-snap-gene-150.25	cysQ	maker-VaccDscaff37-augustus-gene-303.53	4CL-1	0.9507414	0.0035799	0.7714286	0.0723965	0.0152214	0.1204587
maker-VaccDscaff27-snap-gene-56.38	At4g27190	maker-VaccDscaff37-augustus-gene-303.53	4CL-1	0.9532362	0.0032292	0.7714286	0.0723965	0.0146988	0.1204587
maker-VaccDscaff14-snap-gene-66.37	CSNK2B	maker-VaccDscaff37-augustus-gene-303.53	4CL-1	0.9419771	0.0049523	0.8857143	0.0188455	0.0170465	0.0777843
maker-VaccDscaff11-snap-gene-59.16	GCR1	maker-VaccDscaff37-augustus-gene-303.53	4CL-1	0.8983859	0.0149635	0.7714286	0.0723965	0.0271923	0.1204587

maker-VaccDscaff15-augustus-gene-160.24	MYB102	maker-VaccDscaff37-augustus-gene-303.53	4CL-1	0.972806	0.0010992	0.8857143	0.0188455	0.011055	0.0777843
maker-VaccDscaff37-augustus-gene-303.53	4CL-1	snap_masked-VaccDscaff18-processed-gene-237.11	MYB73	0.9438136	0.0046467	0.7714286	0.0723965	0.0165801	0.1204587
maker-VaccDscaff24-snap-gene-213.30	MYB102	maker-VaccDscaff37-augustus-gene-303.53	4CL-1	0.9424475	0.0048731	0.7142857	0.1107872	0.0170052	0.149414
maker-VaccDscaff38-snap-gene-101.15	4CL-2	maker-VaccDscaff47-augustus-gene-152.22	MYO5	0.9830043	0.0004308	0.9428571	0.0048047	0.0093357	0.0355952
maker-VaccDscaff3-snap-gene-76.46	CBP60B	maker-VaccDscaff38-snap-gene-101.15	4CL-2	0.9476944	0.0040323	0.8857143	0.0188455	0.0158513	0.0777843
maker-VaccDscaff25-augustus-gene-325.43	CBP60B	maker-VaccDscaff38-snap-gene-101.15	4CL-2	0.9358855	0.0060342	0.9428571	0.0048047	0.0182877	0.0355952
maker-VaccDscaff38-snap-gene-101.15	4CL-2	maker-VaccDscaff6-augustus-gene-289.20	dgkA	0.9197543	0.0094007	0.9428571	0.0048047	0.0218684	0.0355952
maker-VaccDscaff16-snap-gene-49.29	PPIP5K	maker-VaccDscaff38-snap-gene-101.15	4CL-2	0.9173655	0.0099606	0.7142857	0.1107872	0.0223788	0.149414
maker-VaccDscaff38-augustus-gene-133.15	dgkA	maker-VaccDscaff38-snap-gene-101.15	4CL-2	0.9129967	0.0110251	0.8857143	0.0188455	0.0237359	0.0777843
maker-VaccDscaff38-snap-gene-101.15	4CL-2	maker-VaccDscaff4-snap-gene-355.37	TOC1	0.9905873	0.0001325	0.9428571	0.0048047	0.0084202	0.0355952
maker-VaccDscaff28-snap-gene-111.33	UVR8	maker-VaccDscaff38-snap-gene-101.15	4CL-2	0.8755551	0.0222662	0.8285714	0.0415627	0.0342334	0.1163063
maker-VaccDscaff11-snap-gene-59.16	GCR1	maker-VaccDscaff38-snap-gene-101.15	4CL-2	0.8666306	0.0254949	0.7714286	0.0723965	0.0368583	0.1204587
maker-VaccDscaff24-snap-gene-213.30	MYB102	maker-VaccDscaff38-snap-gene-101.15	4CL-2	0.8823726	0.0199405	0.8285714	0.0415627	0.0320441	0.1163063
maker-VaccDscaff24-augustus-gene-34.41	MYB4	maker-VaccDscaff38-snap-gene-101.15	4CL-2	0.853112	0.0307795	0.7714286	0.0723965	0.0418858	0.1204587
maker-VaccDscaff15-augustus-gene-160.24	MYB102	maker-VaccDscaff38-snap-gene-101.15	4CL-2	0.8511004	0.031606	0.6571429	0.1561749	0.0425863	0.1858537

Supplementary Table S5. Annotation-based screening and expression profiles of bHLH, and WDR candidate

Gene ID	Gene name	FC(treatment/control)	Log2FC(treatment/control)	ExploratoryPvalue	ExploratoryPadjust	Significant	Regulate	Candidate family	GO ID	GO Term	KO ID	KO Name	NR Hit-name	NR Description	Swiss-Prot Hit-name	Swiss-Prot Description	Pfam ID	Domain	Domain Description
maker-VaccDs	BHLH42	0.8858	-0.17	0.0532	0.0658	no	down	bHLH	GO:0006355;GO:0045893;GO:0031542;GO:0016021;GO:0005634;GO:0003677;GO:004698	BP:regulation of transcription, DNA-templated;BP:positive regulation of transcription, DNA-templated;BP:positi	------	------	KAH783	hypothetical protein Vad	sp|A0A2	Transcription factor BHLH42 OS=Acti	PF14215.9;PF00010.29	bHLH-MYC_N;HLH	bHLH-MYC and R2R3-MYB transcription factors N-terminal;Helix-

caff19-augustus-gene-381.30		29	49	63	78					ve regulation of anthocyanin biosynthetic process;CC:integral component of membrane;CC:nucleus;MF:DNA binding;MF:protein dimerization activity;			7881.1	ar_019223 [Vaccinium darrowii]	R6QE26|BHL42_ACTCC;	nidia chinensis var. chinensis OX=1590841 GN=BHLH42 PE=1 SV=1;			loop-helix DNA-binding domain
maker-VaccDscaff4-augustus-	At2g18193	2.398437	1.262095	0.019339	0.034958	yes	up	WDR	GO:0006950;GO:0016887;GO:000552	BP:response to stress;MF:ATPase activity;MF:ATP binding;	------	------	KAG5559857.1	hypothetical protein RHGRI_003222 [Rhodo	sp|Q8GW96|AATP	AAA-ATPase At2g18193 OS=Arabidopsis thaliana OX=3702 GN=At2g18193	PF00004.32;PF14363.9;PF00910.25;PF07728.17;PF20030.2;PF13	AAA;AAA_assoc;RNA_helicase;AAA_5;bpMoxR;AAA_22;AAA	ATPase family associated with various cellular activities;Domain associated at C-terminal with AAA;RNA helicase;AAA domain;MoxR domain in the MoxR-vWA-beta-propeller

gene-29.41														dendrongriersonianum]	3_ARATH;	PE=2 SV=1;	401.9;PF13191.9;PF05673.16	_16;DUF815	ternary systems;AAA domain;AAA ATPase domain;Protein of unknown function
maker-VaccDscaff4-snap-gene-425.50	At1g79990	1.670819	0.7405556	0.0127768	0.0339554	yes	up	WDR	GO:0006886;GO:0016192;GO:0000139;GO:0030117;GO:000519	BP:intracellular protein transport;BP:vesicle-mediated transport;CC:Golgi membrane;CC:membrane coat;MF:structural molecule activity;	K17302	COPB2,SEC27	KAH7857476.1	hypothetical protein Vadar_013156 [Vaccinium darrowii]	sp|Q9CAA0|COB21_ARATH;	Coatomer subunit beta'-1 OS=Arabidopsis thaliana OX=3702 GN=At1g79990 PE=2 SV=2;	PF00400.35;PF20426.1	WD40;NBCH_WD40	WD domain, G-beta repeat;Neurobeachin beta propeller domain

snap_masked-VaccDscaff9-processed-gene-390.25	copb2	1.610249	0.687284	0.000646	0.005571	yes	up	WDR	GO:0006886;GO:0016192;GO:0000139;GO:0030117;GO:000519	BP:intracellular protein transport;BP:vesicle-mediated transport;CC:Golgi membrane;CC:membrane coat;MF:structural molecule activity;	K17302	COPB2,SEC27	GFZ079995.1	coatomer, beta' subunit [Actinidia rufa]	sp|Q54YD8|COPB2_DICDI;	Coatomer subunit beta' OS=Dictyostelium discoideum OX=44689 GN=copb2 PE=3 SV=1;	PF00400.35;PF20426.1	WD40;NBCH_WD40	WD domain, G-beta repeat;Neurobeachin beta propeller domain
maker-VaccDscaff16-augus	PREP	1.595399	0.673917	0.008669	0.027163	yes	up	WDR	GO:0006508;GO:0005737;GO:000425	BP:proteolysis;CC:cytoplasm;MF:serine-type endopeptidase activity;	K01322	PREP	KAH7845840	hypothetical protein Vadar_006620 [Vaccin	sp|P48147|PPCE	Prolyl endopeptidase OS=Homo sapiens OX=9606 GN=PREP PE=1 SV=2;	PF02897.18;PF00326.24	Peptidase_S9_N;Peptidase_S9	Prolyl oligopeptidase, N-terminal beta-propeller domain;Prolyl oligopeptidase family

tus-gene-386.33													.1	ium darrowii]	_HUMAN;				
maker-VaccDscaff43-augustus-gene-25.32	WDR-5	1.546926	0.629404	0.001888	0.004413	yes	up	WDR	GO:0030488;GO:0036265;GO:0008033;GO:0005634;GO:000816	BP:tRNA methylation;BP:RN;BP:tRNA processing;CC:nucleus;MF:methyltransferase activity;	K15443	TRM82,WDR4	KAH7864111.1	hypothetical protein Vadar_025914 [Vaccinium darrowii]	sp|Q54UI3|WDR4_DICDI;	tRNA;	PF00400.35;PF12894.10	WD40;ANAPC4_WD40	WD domain, G-beta repeat;Anaphase-promoting complex subunit 4 WD40 domain
maker-Vacc	PP2A	1.358	0.441	0.009	0.029	yes	up	WDR	GO:0000159;GO:001988	CC:protein phosphatase type 2A complex;MF:protei	K043	PP2	KAH78	hypothetical protein	sp|Q39	Serine/threonine protein phosphatase 2A	PF00400.35	WD40	WD domain, G-beta repeat

Dscaff35-augustus-gene-309.36	B2	433	943	981	318					n phosphatase regulator activity;	54	R2	58426.1	Vadar_023756 [Vaccinium darrowii]	247|2ABB_ARATH;	55 kDa regulatory subunit B beta isoform OS=Arabidopsis thaliana OX=3702 GN=PP2AB2 PE=1 SV=1;				
maker-VaccDscaff377-augustus-gene-	CYPRO4	1.347904	0.430718	0.013004	0.033954	yes	up	WDR	GO:0006952;GO:0005737;GO:0005634;GO:0005635;GO:0003700;GO:000367	BP:defense response;CC:cytoplasm;CC:nucleus;CC:nuclear envelope;MF:transcription factor activity, sequence-specific DNA binding;MF:DNA binding;	------	------	KAH7833879.1	hypothetical protein Vadar_010731 [Vaccinium darrowii]	sp|P40781|CYPR4_CYN	Protein CYPRO4 OS=Cynara cardunculus OX=4265 GN=CYPRO4 PE=2 SV=1;	PF08553.13	VID27	VID27 C-terminal WD40-like domain	

0.26																			
maker-VaccDscaff29-augustus-gene-312.21	NMAT3	1.333026	0.414704	0.081465	0.089043	no	up	WDR	GO:0006952;GO:0006355;GO:0005829;GO:004353	BP:defense response;BP:regulation of transcription, DNA-templated;CC:cytosol;MF:ADP binding;	------	------	KAH7854588.1	hypothetical protein Vadar_015662 [Vaccinium darrowii]	CA;sp|Q9LZA5|NMAT3_ARATH;	Nuclear intron maturase 3, mitochondrial OS=Arabidopsis thaliana OX=3702 GN=NMAT3 PE=3 SV=2;	PF00931.25;PF01348.24;PF00078.30;PF00400.35;PF13401.9;PF13855.9;PF13238.9;PF13191.9	NB-ARC;Intron_maturas2;RVT_1;WD40;AAA_22;LRR_8;AAA_18;AAA_16	NB-ARC domain;Type II intron maturase;Reverse transcriptase;WD domain, G-beta repeat;AAA domain;Leucine rich repeat;AAA domain;AAA ATPase domain
maker-VaccDscaf	TPR3	1.32033	0.40009	0.07274	0.08814	no	up	WDR	GO:0006952;GO:0006355;GO:0005829;GO:004353	BP:defense response;BP:regulation of transcription, DNA-templated;CC:cytosol;MF:ADP binding;	------	------	KAF71447	hypothetical protein RHSIM	sp|Q84JM4|	Topless-related protein 3 OS=Arabidopsis thaliana OX=370	PF00931.25;PF13855.9;PF00400.35;PF	NB-ARC;LRR_8;WD40;ANAPC4_	NB-ARC domain;Leucine rich repeat;WD domain, G-beta repeat;Anaphase-promoting complex

f26-augustus-gene-326.29		81	55	63	26								2000.1	_Rhsim03G0040200 [Rhododendron simsii]	TPR3_ARATH;	2 GN=TPR3 PE=1 SV=1;	12894.10	WD40	subunit 4 WD40 domain
maker-VaccDscaff40-snap-gene-251.28	wdr11	1.313451	0.393362	0.019314	0.034958	yes	up	WDR	------	------	K24260	WDR11	KAH7850159.1	hypothetical protein Vadar_028653 [Vaccinium darrowii]	sp|F1QEB7|WDR11_DANRE;	WD repeat-containing protein 11 OS=Danio rerio OX=7955 GN=wdr11 PE=2 SV=1;	------	------	------

maker-VaccDscaff18-augustus-gene-205.28	LUG	1.279177	0.355216	0.026107	0.042311	yes	up	WDR	GO:000371	MF:transcription corepressor activity;	------	------	KAH7845325.1	hypothetical protein Vadar_000665 [Vaccinium darrowii]	sp|Q9FUY2|LEUNG_ARATH;	Transcriptional corepressor LEUNIG OS=Arabidopsis thaliana OX=3702 GN=LUG PE=1 SV=2;	PF00400.35;PF12894.10	WD40;ANAPC4_WD40	WD domain, G-beta repeat;Anaphase-promoting complex subunit 4 WD40 domain
maker-VaccDscaff2-augus	CYPRO4	1.273354	0.348633	0.021168	0.036848	yes	up	WDR	GO:0006952;GO:0005737;GO:0005634;GO:0005635;GO:0003700;GO:000367	BP:defense response;CC:cytoplasm;CC:nucleus;CC:nuclear envelope;MF:transcription factor activity, sequence-specific DNA binding;MF:DNA binding;	-----	-----	KAH7833879	hypothetical protein Vadar_010731 [Va	sp|P40781|CYP	Protein CYPRO4 OS=Cynara cardunculus OX=4265 GN=CY	PF08553.13	VID27	VID27 C-terminal WD40-like domain

tus-gene-412.35													.1	ccinium darrowii]	R4_CYNCA;	PRO4 PE=2 SV=1;			
snap_masked-VaccDscaff18-processed-gene-73.7	WDR-6	0.890599	-0.16715	0.051192	0.065028	no	down	WDR	GO:001602	CC:integral component of membrane;	------	------	KAH7847398.1	hypothetical protein Vadar_025614 [Vaccinium darrowii]	sp|Q9Y7K5|YGI3_SCHPO;	Uncharacterized WD repeat-containing protein C2A9.03 OS=Schizosaccharomyces pombe;	PF12894.10;PF00400.35;PF20426.1	ANAPC4_WD40;WD40;NBCH_WD40	Anaphase-promoting complex subunit 4 WD40 domain;WD domain, G-beta repeat;Neurobeachin beta propeller domain

maker-VaccDscaff36-snap-gene-244.37	AMBRA1	0.890146	-0.167889	0.035605	0.052288	no	down	WDR	GO:0006914;GO:004323	BP:autophagy;CC:intracellular membrane-bounded organelle;	K17985	AMBRA1	KAH7856323.1	hypothetical protein Vadar_000076 [Vaccinium darrowii]	sp|Q9C0C7|AMRA1_HUMAN;sp	Activating molecule in BECN1-regulated autophagy protein 1 OS=Homo sapiens OX=9606 GN=AMBRA1 PE=1 SV=2;	PF00400.35;PF12894.10	WD40;ANAPC4_WD40	WD domain, G-beta repeat;Anaphase-promoting complex subunit 4 WD40 domain
maker-VaccDscaff32-sna	CSTF50	0.887942	-0.17146	0.107255	0.114568	no	down	WDR	GO:0031124;GO:000584	BP:mRNA 3'-end processing;CC:mRNA cleavage stimulating factor complex;	K14406	CSTF1	PSS08003.1	Cleavage stimulation factor subunit like	sp|Q8L4J2|CTF	Cleavage stimulation factor subunit 50 OS=Arabidopsis thaliana OX=370	PF00400.35;PF20426.1	WD40;NBCH_WD40	WD domain, G-beta repeat;Neurobeachin beta propeller domain

p-gene-150.28														[Actinidia chinensis var. chinensis]	50_ARATH;	2 GN=CSTF50 PE=1 SV=1;			
maker-VaccDscaff29-augustus-gene-123.23	STXBP5L	0.885165	-0.17598	0.004697	0.018398	yes	down	WDR	GO:0009555;GO:0017157;GO:0005737;GO:0005802;GO:0005770;GO:0005886;GO:0000149;GO:0005096;GO:0019905;GO:004515	BP:pollen development;BP:regulation of exocytosis;CC:cytoplasm;CC:trans-Golgi network;CC:late endosome;CC:plasma membrane;MF:SNARE binding;MF:GTPase activator activity;MF:syntaxin binding;MF:myosin II binding;	K08518	STXBP5,SRO7_77	KAH7854128.1	hypothetical protein Vadar_010404 [Vaccinium darrowii]	sp|Q9Y2K9|STB5L_HUMAN;	Syntaxin-binding protein 5-like OS=Homo sapiens OX=9606 GN=STXBP5L PE=1 SV=2;	PF12894.10;PF00957.24	ANAPC4_WD40;Synaptobrevin	Anaphase-promoting complex subunit 4 WD40 domain;Synaptobrevin

augustus_masked-VaccDscaff46-processed-gene-162.3	ADHIII	0.8832688	-0.179008	0.016022	0.0349588	yes	down	WDR	GO:000573	CC:cytoplasm;	K22382	WDR26	KAG5567349.1	hypothetical protein RHGRI_002793 [Rhododendron griersonianum]	sp|A2XAZ3|ADHX_ORYSI;	Alcohol dehydrogenase class-3 OS=Oryza sativa subsp. indica OX=39946 GN=ADHIII PE=3 SV=1;	PF08240.15;PF00107.29;PF00400.35;PF20426.1	ADH_N;ADH_zinc_N;WD40;NBCH_WD40	Alcohol dehydrogenase GroES-like domain;Zinc-binding dehydrogenase;WD domain, G-beta repeat;Neurobeachin beta propeller domain
maker-VaccDscaff40-au	WDR-4	0.869117	-0.202229	0.0717077	0.0814226	no	down	WDR	GO:001602	CC:integral component of membrane;	------	------	KAG5544344	hypothetical protein RHGRI_016930	sp|Q9Y7K5|YG	Uncharacterized WD repeat-containing protein C2A9.03 OS=Schizosaccha	PF12894.10;PF00400.35;PF20426.1	ANAPC4_WD40;WD40;NBCH_WD40	Anaphase-promoting complex subunit 4 WD40 domain;WD domain, G-beta repeat;Neurobeachin beta

gustus-gene-91.21													.1	[Rhododendron griersonianum]	I3_SCHPO;	romyces pombe;			propeller domain
maker-VaccDscaff16-snap-gene-263.25	ATG18H	0.864995	-0.209924	0.001608	0.010794	yes	down	WDR	GO:0006468;GO:0006914;GO:0042594;GO:0005524;GO:000467	BP:protein phosphorylation;BP:autophagy;BP:response to starvation;MF:ATP binding;MF:protein kinase activity;	------	------	KAF7138777.1	hypothetical protein RHSIM_Rhsim07G0178500 [Rhododendron simsii]	sp|Q8H1Q5|ATG18H_ARATH;	Autophagy-related protein 18h OS=Arabidopsis thaliana OX=3702 GN=ATG18H PE=2 SV=1;	PF12490.11;PF00400.35	BCAS3;WD40	Breast carcinoma amplified sequence 3;WD domain, G-beta repeat

maker-VaccDscaff30-snap-gene-66.27	VPS15	0.859691	-0.218111	0.003999	0.018398	yes	down	WDR	GO:0006468;GO:0030242;GO:0016236;GO:0006623;GO:0045324;GO:0005770;GO:0016020;GO:0071561;GO:0034272;GO:0034271;GO:0004674;GO:000552	BP:protein phosphorylation;BP:pexophagy;BP:macroautophagy;BP:protein targeting to vacuole;BP:late endosome to vacuole transport;CC:late endosome;CC:membrane;CC:nucleus-vacuole junction;CC:phosphatidylinositol 3-kinase complex, class III, type II;CC:phosphatidylinositol 3-kinase complex, class III, type I;MF:protein serine/threonine kinase activity;MF:ATP binding;	K08333	PIK3R4, VPS15	KAH7851437.1	hypothetical protein Vadar_011569 [Vaccinium darrowii]	sp|Q9M0E5|VPS15_ARATH;	Serine/threonine-protein kinase VPS15 OS=Arabidopsis thaliana OX=3702 GN=VPS15 PE=1 SV=1;	PF00069.28;PF00400.35;PF02985.25;PF07714.20;PF20426.1	Pkinase;WD40;HEAT;PK_Tyr_Ser-Thr;NBCH_WD40	Protein kinase domain;WD domain, G-beta repeat;HEAT repeat;Protein tyrosine and serine/threonine kinase;Neurobeachin beta propeller domain
maker-VaccDs	SPA3	0.8504	-0.233	0.1792	0.1792	no	down	WDR	GO:0006468;GO:0009640;GO:0016021;GO:0005524;GO:000467	BP:protein phosphorylation;BP:photomorphogenesis;CC:integral component of membrane;MF:ATP	------	------	KAH786	hypothetical protein Vad	sp|Q9LJ	Protein SPA1-RELATED 3 OS=Arabidopsis	PF00400.35;PF00069.28	WD40;Pkinase	WD domain, G-beta repeat;Protein kinase domain

caff607-snap-gene-0.11		59	69	73	73					binding;MF:protein kinase activity;			0711.1	ar_017096 [Vaccinium darrowii]	R3|SPA3_ARATH;	thaliana OX=3702 GN=SPA3 PE=1 SV=1;			
maker-VaccDscaff207-augustus-gene-1.31	WDR-3	0.831992	-0.265536	0.037587	0.052288	no	down	WDR	------	------	------	------	KAH7844776.1	hypothetical protein Vadar_031566 [Vaccinium darrowii]	sp|Q9Y7K5|YGI3_SCHP	Uncharacterized WD repeat-containing protein C2A9.03 OS=Schizosaccharomyces pombe;	PF00400.35	WD40	WD domain, G-beta repeat

maker-VaccDscaff16-augustus-gene-322.38	WDR-2	0.828414	-0.271558	0.017977	0.034958	yes	down	WDR	GO:001602	CC:integral component of membrane;	------	------	KAH7847398.1	hypothetical protein Vadar_025614 [Vaccinium darrowii]	O;sp|Q9Y7K5|YGI3_SCHPO;	Uncharacterized WD repeat-containing protein C2A9.03 OS=Schizosaccharomyces pombe;	PF00400.35	WD40	WD domain, G-beta repeat
augustus_masked-VaccDscaf	WDR-1	0.802765	-0.316995	6.45E-05	0.003033	yes	down	WDR	------	------	------	------	KAH7833782	hypothetical protein Vadar_009664 [Va	------	------	PF07676.15	PD40	WD40-like Beta Propeller Repeat

f2-processed-gene-297.5													.1	ccinium darrowii]						
maker-VaccDscaff21-snap-gene-11.34	XI-K	0.777122	-0.363779	0.046926	0.063016	no	down	WDR	GO:0048467;GO:0060151;GO:0030050;GO:0051301;GO:0090436;GO:0048768;GO:0030048;GO:0051646;GO:0051645;GO:0007015;GO:0010154;GO:0010091;GO:0015629;GO:0030133;GO:0016459;GO:0035619;GO:0000146;GO:0003779;GO:0003774;GO:0005524;GO:0051015;GO:000551	BP:gynoecium development;BP:peroxisome localization;BP:vesicle transport along actin filament;BP:cell division;BP:leaf pavement cell development;BP:root hair cell tip growth;BP:actin filament-based movement;BP:mitochondrion localization;BP:Golgi localization;BP:actin filament organization;BP:fruit	K10357	MYO5	KAG5559159.1	hypothetical protein RHGRI_008919 [Rhododendron griersonianum]	sp|F4K5J1|MYO17_ARATH;	Myosin-17 OS=Arabidopsis thaliana OX=3702 GN=XI-K PE=1 SV=2;	PF00063.24;PF00400.35;PF01843.22;PF00612.30;PF12894.10;PF02736.22;PF20426.1	Myosin_head;WD40;DIL;IQ;ANAPC4_WD40;Myosin_N;NBCH_WD40	Myosin head;WD domain, G-beta repeat;DIL domain;IQ calmodulin-binding motif;Anaphase-promoting complex subunit 4 WD40 domain;Myosin N-terminal SH3-like domain;Neurobeachin beta propeller domain

										development;BP:trichome branching;CC:actin cytoskeleton;CC:transport vesicle;CC:myosin complex;CC:root hair tip;MF:microfilament motor activity;MF:actin binding;MF:motor activity;MF:ATP binding;MF:actin filament binding;MF:calmodulin binding;									
maker-VaccDscaff31-augustus-	ATG18H	0.774495	-0.36867	0.067452	0.079256	no	down	WDR	GO:0042594;GO:0006914;GO:0006468;GO:0005524;GO:000467	BP:response to starvation;BP:autophagy;BP:protein phosphorylation;MF:ATP binding;MF:protein kinase activity;	------	------	KAF7138777.1	hypothetical protein RHSIM_Rhsim07G0178500	sp|Q8H1Q5|ATG18H	Autophagy-related protein 18h OS=Arabidopsis thaliana OX=3702 GN=ATG18H	PF12490.11;PF00400.35	BCAS3;WD40	Breast carcinoma amplified sequence 3;WD domain, G-beta repeat

gene-209.16														[Rhododendron simsii]	_ARATH;	PE=2 SV=1;			
maker-VaccDscaff42-augustus-gene-269.54	VPS15	0.763464	-0.389337	0.004357	0.018398	yes	down	WDR	GO:0006468;GO:0030242;GO:0016236;GO:0006623;GO:0045324;GO:0005770;GO:0016020;GO:0071561;GO:0034272;GO:0034271;GO:0004674;GO:000552	BP:protein phosphorylation;BP:pexophagy;BP:macroautophagy;BP:protein targeting to vacuole;BP:late endosome to vacuole transport;CC:late endosome;CC:membrane;CC:nucleus-vacuole junction;CC:phosphatidylinositol 3-kinase complex, class III, type II;CC:phosphatidylinositol 3-kinase complex, class III, type I;MF:protein serine/threonine kinase	K08333	PIK3R4, VPS15	KAH7851437.1	hypothetical protein Vadar_011569 [Vaccinium darrowii]	sp|Q9M0E5|VPS15_ARATH;	Serine/threonine-protein kinase VPS15 OS=Arabidopsis thaliana OX=3702 GN=VPS15 PE=1 SV=1;	PF00069.28;PF02985.25;PF00400.35;PF07714.20;PF20426.1	Pkinase;HEAT;WD40;PK_Tyr_Ser-Thr;NBCH_WD40	Protein kinase domain;HEAT repeat;WD domain, G-beta repeat;Protein tyrosine and serine/threonine kinase;Neurobeachin beta propeller domain

										activity;MF:ATP binding;									
maker-VaccDscaf105-snap-gene-2.30	Vps8	0.717431	-0.479099	0.114897	0.120003	no	down	WDR	GO:0016192;GO:0009555;GO:0006623;GO:0034058;GO:0030897;GO:0005634;GO:0005770;GO:011016	BP:vesicle-mediated transport;BP:pollen development;BP:protein targeting to vacuole;BP:endosomal vesicle fusion;CC:HOPS complex;CC:nucleus;CC:late endosome;CC:cellular anatomical entity;	K20178	VPS8	KAH7834330.1	hypothetical protein Vadar_014963 [Vaccinium darrowii]	sp|Q0P5W1|VPS8_MOUSE;	Vacuolar protein sorting-associated protein 8 homolog OS=Mus musculus OX=10090 GN=Vps8 PE=1 SV=1;	PF12816.10;PF12894.10	Vps8;ANAPC4_WD40	Golgi CORVET complex core vacuolar protein 8;Anaphase-promoting complex subunit 4 WD40 domain
maker-VaccDscaf9-snap-	TPR1	0.713752	-0.4865	0.055286	0.066627	no	down	WDR	GO:0009791;GO:0006355;GO:0048608;GO:004836	BP:post-embryonic development;BP:regulation of transcription, DNA-templated;BP:reproductive structure development;BP:shoot system development;	------	------	KAH7858028	hypothetical protein Vadar_019229 [Va	sp|Q0WV90|TPP	Topless-related protein 1 OS=Arabidopsis thaliana OX=3702 GN=TP	PF00400.35;PF12894.10;PF17814.4	WD40;ANAPC4_WD40;LisH_TPL	WD domain, G-beta repeat;Anaphase-promoting complex subunit 4 WD40 domain;LisH-like

gene-380.21													.1	ccinium darrowii]	R1_ARATH;	R1 PE=1 SV=3;			dimerisation domain
maker-VaccDscaff26-augustus-gene-8.15	VIP3	0.678011	-0.560662	0.033683	0.051506	no	down	WDR	GO:0051568;GO:0016593;GO:000563	BP:histone H3-K4 methylation;CC:Cdc73/Paf1 complex;CC:nucleus;	K12602	WDR61,REC14,SKI8	KAH7853884.1	hypothetical protein Vadar_007632 [Vaccinium darrowii]	sp|Q9SZQ5|VIP3_ARATH;	WD repeat-containing protein VIP3 OS=Arabidopsis thaliana OX=3702 GN=VIP3 PE=1 SV=1;	PF00400.35;PF12894.10;PF20426.1;PF08662.14	WD40;ANAPC4_WD40;NBCH_WD40;eIF2A	WD domain, G-beta repeat;Anaphase-promoting complex subunit 4 WD40 domain;Neurobeachin beta propeller domain;Eukaryotic translation initiation factor eIF2A
maker-	BUB	0.6	-0.	0.0	0.0	yes	do	WDR	------	------	K02	BU	KAH	hypothetical	sp|Q	Mitotic checkpoint	PF00400.35	WD40	WD domain, G-beta repeat

VaccDscaff2-augustus-gene-402.41	3.2	74757	56756	16772	34958		wn				180	B3	7835839.1	protein Vadar_030385 [Vaccinium darrowii]	9C701|BUB32_ARATH;	protein BUB3.2 OS=Arabidopsis thaliana OX=3702 GN=BUB3.2 PE=2 SV=1;			
maker-VaccDscaff1-snap-gene-37	COP1	0.538776	-0.892224	0.007952	0.026696	yes	down	WDR	GO:0046872;GO:006163	MF:metal ion binding;MF:ubiquitin protein ligase activity;	K10143	RFWD2, COP1	KAH7842985.1	hypothetical protein Vadar_011322 [Vaccinium darr	sp|P43254|COP1_A	E3 ubiquitin-protein ligase COP1 OS=Arabidopsis thaliana OX=3702 GN=COP1 PE=1 SV=2;	PF00400.35;PF13923.9;PF00097.28;PF13920.9;PF17005.8;PF13639.9	WD40;zf-C3HC4_2;zf-C3HC4;zf-C3HC4_3;WD40_like;zf-	WD domain, G-beta repeat;Zinc finger, C3HC4 type;Zinc finger, C3HC4 type;Zinc finger, C3HC4 type;WD40-like domain;Ring finger domain;RING-type zinc-finger;zinc-

3.47														owii]	RATH;		;PF13445.9;PF14634.9	RING_2;zf-RING_UBOX;zf-RING_5	RING finger domain
maker-VaccDscaff15-augustus-gene-343.35	bHLH30	2.208633	1.143154	0.000451	0.005571	yes	up	bHLH	GO:0005634;GO:0003700;GO:004698	CC:nucleus;MF:transcription factor activity, sequence-specific DNA binding;MF:protein dimerization activity;	------	------	KAH7836416.1	hypothetical protein Vadar_000995 [Vaccinium darrowii]	sp|Q9S7Y1|BH030_ARATH;	Transcription factor bHLH30 OS=Arabidopsis thaliana OX=3702 GN=BHLH30 PE=1 SV=1;	PF00010.29	HLH	Helix-loop-helix DNA-binding domain

maker-VaccDscaff11-augustus-gene-31.20	bHLH130	1.918969	0.940331	0.018238	0.034958	yes	up	bHLH	GO:0006355;GO:0016021;GO:0005634;GO:0046983;GO:000367	BP:regulation of transcription, DNA-templated;CC:integral component of membrane;CC:nucleus;MF:protein dimerization activity;MF:DNA binding;	------	------	KAH7838264.1	hypothetical protein Vadar_024191 [Vaccinium darrowii]	sp|Q66GR3|BH130_ARATH;	Transcription factor bHLH130 OS=Arabidopsis thaliana OX=3702 GN=BHLH130 PE=1 SV=1;	PF00010.29	HLH	Helix-loop-helix DNA-binding domain
snap_masked-VaccDscaff43	MYC2	1.32233	0.403082	0.002492	0.013016	yes	up	bHLH	GO:0006979;GO:0016021;GO:0005634;GO:0005886;GO:0020037;GO:0140825;GO:0003700;GO:0003677;GO:0046983;GO:004687	BP:response to oxidative stress;CC:integral component of membrane;CC:nucleus;CC:plasma membrane;MF:heme binding;MF:old GO;MF:transcription factor activity,	K13422	MYC2	KAH7863800	hypothetical protein Vadar_022175 [Va	sp|A0A3Q7HR	Transcription factor MYC2 OS=Solanum lycopersicum OX=4081	PF14215.9;PF00010.29;PF18035.4	bHLH-MYC_N;HLH;Bap31_Bap29_C	bHLH-MYC and R2R3-MYB transcription factors N-terminal;Helix-loop-helix DNA-binding domain;Bap31/Bap29

-processed-gene-196.19										sequence-specific DNA binding;MF:DNA binding;MF:protein dimerization activity;MF:metal ion binding;			.1	ccinium darrowii]	Z6|MYC2_SOLLC;sp|	GN=MYC2 PE=1 SV=1;			cytoplasmic coiled-coil domain
augustus_masked-VaccDscaff22-processed-ge	MTB1	1.29913	0.377546	0.005904	0.021344	yes	up	bHLH	GO:0005634;GO:0003700;GO:0046983;GO:000367	CC:nucleus;MF:transcription factor activity, sequence-specific DNA binding;MF:protein dimerization activity;MF:DNA binding;	------	------	KAH7862908.1	hypothetical protein Vadar_010989 [Vaccinium darrowii]	A0A3Q7ELQ2|MTB1_	Transcription factor MTB1 OS=Solanum lycopersicum OX=4081 GN=MTB1 PE=1 SV=1;	PF14215.9;PF00010.29	bHLH-MYC_N;HLH	bHLH-MYC and R2R3-MYB transcription factors N-terminal;Helix-loop-helix DNA-binding domain

ne-4.3																			
maker-VaccDscaff34-augustus-gene-66.24	BHLH004	0.908173	-0.138996	0.050955	0.065028	no	down	bHLH	GO:0006355;GO:0005634;GO:0003700;GO:0046983;GO:000367	BP:regulation of transcription, DNA-templated;CC:nucleus;MF:transcription factor activity, sequence-specific DNA binding;MF:protein dimerization activity;MF:DNA binding;	------	------	AOY34375.1	transcription factor BHLH004, partial [Vaccinium corymbosum]	SOLLC;sp|Q93W88|BH137_ARATH;	Transcription factor bHLH137 OS=Arabidopsis thaliana OX=3702 GN=BHLH137 PE=1 SV=1;	PF00010.29	HLH	Helix-loop-helix DNA-binding domain
maker-Va	BHLH	0.88	-0.1	0.00	0.00	yes	down	bHLH	GO:0006355;GO:0005634;GO:0003700;GO:004698	BP:regulation of transcription, DNA-templated;CC:nucleus;MF:transcription	----	----	AOY3	transcription	sp|Q9	Transcription factor bHLH13	PF00010.29	HLH	Helix-loop-helix DNA-binding domain

ccDscaff50-augustus-gene-11.22	004	5089	761	0729	571					factor activity, sequence-specific DNA binding;MF:protein dimerization activity;	--	--	4375.1	factor BHLH004, partial [Vaccinium corymbosum]	3W88|BH137_ARATH;	7 OS=Arabidopsis thaliana OX=3702 GN=BHLH137 PE=1 SV=1;			
maker-VaccDscaff32-augustus-ge	PRE6	0.865634	-0.208171	0.014248	0.034893	yes	down	bHLH	GO:0006355;GO:0040008;GO:0005634;GO:004698	BP:regulation of transcription, DNA-templated;BP:regulation of growth;CC:nucleus;MF:protein dimerization activity;	------	------	KAH7852738.1	hypothetical protein Vadar_028568 [Vaccinium darr	sp|Q8GW32|PRE6_A	Transcription factor PRE6 OS=Arabidopsis thaliana OX=3702 GN=PRE6 PE=1 SV=1;	PF00010.29;PF19220.3	HLH; Crescentin	Helix-loop-helix DNA-binding domain;Crescentin protein

ne-287.19														owii]	RATH;				
snap_masked-VaccDscaff13-processed-gene-48.41	SCRM	0.862324	-0.21337	0.167118	0.170751	no	down	bHLH	GO:0006355;GO:0016021;GO:0005634;GO:0004523;GO:0003676;GO:004698	BP:regulation of transcription, DNA-templated;CC:integral component of membrane;CC:nucleus;MF:RNA-DNA hybrid ribonuclease activity;MF:nucleic acid binding;MF:protein dimerization activity;	------	------	KAH7852736.1	hypothetical protein Vadar_028552 [Vaccinium darrowii]	sp|Q9LSE2|ICE1_ARATH;	Transcription factor ICE1 OS=Arabidopsis thaliana OX=3702 GN=SCRM PE=1 SV=1;	PF00010.29	HLH	Helix-loop-helix DNA-binding domain
maker-VaccDs	BHLH0	0.80788	-0.3077	0.01448	0.03448	yes	down	bHLH	GO:0006355;GO:0005634;GO:0003700;GO:004698	BP:regulation of transcription, DNA-templated;CC:nucleus;MF:transcription factor activity, sequence-specific	------	------	AOY343	transcription factor	sp|Q93W	Transcription factor bHLH137 OS=Ara	PF00010.29	HLH	Helix-loop-helix DNA-binding domain

caff27-snap-gene-278.27	04	98	75	48	93					DNA binding;MF:protein dimerization activity;			75.1	BHLH004, partial [Vaccinium corymbosum]	88|BHLH137_ARATH;	bidopsis thaliana OX=3702 GN=BHLH137 PE=1 SV=1;			
maker-VaccDscaff30-augustus-gene-30	SCRM	0.789743	-0.340555	0.002292	0.013016	yes	down	bHLH	GO:0006355;GO:0005634;GO:0046983;GO:0004523;GO:000367	BP:regulation of transcription, DNA-templated;CC:nucleus;MF:protein dimerization activity;MF:RNA-DNA hybrid ribonuclease activity;MF:nucleic acid binding;	------	------	KAH7852736.1	hypothetical protein Vadar_028552 [Vaccinium darrowii]	sp|Q9LSE2|ICE1_ARA	Transcription factor ICE1 OS=Arabidopsis thaliana OX=3702 GN=SCRM PE=1 SV=1;	PF00010.29	HLH	Helix-loop-helix DNA-binding domain

4.40															TH;				
maker-VaccDscaff30-augustus-gene-327.30	PRE6	0.77972	-0.358977	0.000669	0.005571	yes	down	bHLH	GO:0006355;GO:0040008;GO:0005634;GO:004698	BP:regulation of transcription, DNA-templated;BP:regulation of growth;CC:nucleus;MF:protein dimerization activity;	------	------	KAH7852738.1	hypothetical protein Vadar_028568 [Vaccinium darrowii]	sp|Q8GW32|PRE6_ARATH;	Transcription factor PRE6 OS=Arabidopsis thaliana OX=3702 GN=PRE6 PE=1 SV=1;	PF00010.29;PF19220.3	HLH; Crescentin	Helix-loop-helix DNA-binding domain;Crescentin protein
maker-VaccDscaff34	At1g189	0.674551	-0.568099	0.037826	0.052288	no	down	bHLH	GO:0006355;GO:0048046;GO:0005634;GO:0046983;GO:003014	BP:regulation of transcription, DNA-templated;CC:apoplast;CC:nucleus;MF:protein dimerization activity;MF:manganese ion binding;	------	------	KAH786651	hypothetical protein Vadar_0022	sp|Q9LMC9|	Germin-like protein subfamily T member 2 OS=Ara	PF00010.29	HLH	Helix-loop-helix DNA-binding domain

-augustus-gene-120.28	80												01.1	66 [Vaccinium darrowii]	GLT2_ARATH;	bidopsis thaliana OX=3702 GN=At1g18980 PE=2 SV=1;			
maker-VaccDscaff22-augustus-gene-46.34	BHLH010	0.626691	-0.674417	0.025073	0.042087	yes	down	bHLH	GO:0006355;GO:0016021;GO:0005634;GO:0046983;GO:000367	BP:regulation of transcription, DNA-templated;CC:integral component of membrane;CC:nucleus;MF:protein dimerization activity;MF:DNA binding;	------	------	AOY34381.1	transcription factor BHLH010, partial [Vaccinium corymbosum]	sp|Q8GZ38|UNE10_ARAT	Transcription factor UNE10 OS=Arabidopsis thaliana OX=3702 GN=UNE10 PE=2 SV=1;	PF00010.29	HLH	Helix-loop-helix DNA-binding domain

maker-VaccDscaff17-augustus-gene-313.25	BHLH004	0.411382	-1.28145	0.033972	0.051506	no	down	bHLH	GO:0006355;GO:0005634;GO:0003700;GO:004698	BP:regulation of transcription, DNA-templated;CC:nucleus;MF:transcription factor activity, sequence-specific DNA binding;MF:protein dimerization activity;	-----	-----	AOY34375.1	transcription factor BHLH004, partial [Vaccinium corymbosum]	H;sp|Q93W88|BH137_ARATH;	Transcription factor bHLH137 OS=Arabidopsis thaliana OX=3702 GN=BHLH137 PE=1 SV=1;	PF00010.29	HLH	Helix-loop-helix DNA-binding domain

Supplementary Table S6. Cis-acting elements of the VcUFGT promoter

Gene name	Cis-acting elements	Numbers	Sequence	Functions
maker-VaccDscaff19-snap-gene-64.36	TC-rich repeats	1	GTTTTCTTAC	cis-acting element involved in defense and stress responsiveness
	LTR	1	CCGAAA	cis-acting element involved in low-temperature responsiveness
	ARE	6	AAACCA	cis-acting regulatory element essential for the anaerobic induction
	ACE	1	CTAACGTATT	cis-acting regulatory element involved in light responsiveness
	GA-motif	1	ATAGATAA	
	GATA-motif	1	AAGGATAAGG	
	I-box	1	gGATAAGGTG	
	Sp1	1	GGGCGG	
	TCT-motif	1	TCTTAC	
	Box 4	2	ATTAAT	

	CGTCA-motif/TGACG-motif	10	CGTCA/TGACG	cis-acting regulatory element involved in the MeJA-responsiveness
	CAAT-box	40	CAAAT/CAAT/CCAAT	common cis-acting element in promoter and enhancer regions
	TATA-box	20	TACAAAA	core promoter element around -30 of transcription start
	GC-motif	1	CCCCCG	enhancer-like element involved in anoxic specific inducibility
	AuxRE	1	TGTCTCAATAAG	part of an auxin-responsive element
	AAGAA-motif	1	gGTAAAGAAA	
	AT~TATA-box	1	TATATA	
	as-1	5	TGACG	
	MYB	8	CAACTG/TAACCA/CAACCA/CAACAG/TAACCA	
	MYC	5	CATTTG	
	TATA	1	TATAAAAT	
	TCA	1	TCATCTTCAT	
	STRE	2	AGGGG	
	Unnamed__1	3	CGTGG	
	Unnamed__2	1	CCCCGG	
	Unnamed__4	18	CTCC	
	TC-rich repeats	3	GTTTTCTTAC/ATTCTCTAAC	cis-acting element involved in defense and stress responsiveness
	MRE	1	AACCTAA	
maker-VaccDscaff38-augustus-gene-9.29	Box II	1	TGGTAATAA	
	TCCC-motif	2	TCTCCCT	cis-acting element involved in light responsiveness
	ACE	3	GACACGTATG/CTAACGTATT	
	GT1-motif	3	GGTTAA/GTGTGTGAA	

Box 4	3	ATTAAT	
G-box	6	CACGTG/TACGTG/TCCACATGGCA	
ABRE	6	ACGTG/CGCACGTGTC/CACGTG/TACGTGTC	cis-acting element involved in the abscisic acid responsiveness
ARE	5	AAACCA	cis-acting regulatory element essential for the anaerobic induction
CGTCA-motif/TGACG-motif	2	CGTCA/TGACG	cis-acting regulatory element involved in the MeJA-responsiveness
CAT-box	1	GCCACT	cis-acting regulatory element related to meristem expression
CAAT-box	31	CAAAT/CCAAT/TGCCAAC/CAAT	common cis-acting element in promoter and enhancer regions
TATA-box	26	TATA/TACAAAA/ATATAA/TATTTAAA/ccTATAAAaa/ATATAT/TATATA/ATTATA/TATATAA/TATAAAA/TATAAA/TATAA	core promoter element around -30 of transcription start
GC-motif	1	CCCCCG	enhancer-like element involved in anoxic specific inducibility
MBS	1	CAACTG	MYB binding site involved in drought-inducibility
CCAAT-box	1	CAACGG	MYBHv1 binding site
STRE	1	AGGGG	
Unnamed__16	1	GCTGCCCGTC	
Unnamed__2	1	AACCTAACCT	
WRE3	1	CCACCT	
AT~TATA-box	2	TATATA	
CARE	2	CAACTCAC	
TATA	2	TATAAAAT	
W box	2	TTGACC	

Unnamed__1	3	CGTGG
MYB	5	CAACTG/CAACCA/CCGTTG
MYC	5	CATGTG/TCTCTTA
Unnamed__4	20	CTCC
AAGAA-motif	1	gGTAAAGAAA
ABRE3a	1	TACGTG
ABRE4	1	CACGTA
as-1	1	TGACG
AT~ABRE	1	TACGTGTC
